# Climate Change May Alter Breeding Ground Distributions of Eastern Migratory Monarchs (*Danaus plexippus*) via Range Expansion of *Asclepias* Host Plants

**DOI:** 10.1371/journal.pone.0118614

**Published:** 2015-02-23

**Authors:** Nathan P. Lemoine

**Affiliations:** Department of Biological Sciences, Florida International University, Miami, Florida, United States of America; USDA-Agricultural Research Service, UNITED STATES

## Abstract

Climate change can profoundly alter species’ distributions due to changes in temperature, precipitation, or seasonality. Migratory monarch butterflies (*Danaus plexippus*) may be particularly susceptible to climate-driven changes in host plant abundance or reduced overwintering habitat. For example, climate change may significantly reduce the availability of overwintering habitat by restricting the amount of area with suitable microclimate conditions. However, potential effects of climate change on monarch northward migrations remain largely unknown, particularly with respect to their milkweed (*Asclepias spp*.) host plants. Given that monarchs largely depend on the genus *Asclepias* as larval host plants, the effects of climate change on monarch northward migrations will most likely be mediated by climate change effects on *Asclepias*. Here, I used MaxEnt species distribution modeling to assess potential changes in *Asclepias* and monarch distributions under moderate and severe climate change scenarios. First, *Asclepias* distributions were projected to extend northward throughout much of Canada despite considerable variability in the environmental drivers of each individual species. Second, *Asclepias* distributions were an important predictor of current monarch distributions, indicating that monarchs may be constrained as much by the availability of *Asclepias* host plants as environmental variables *per se*. Accordingly, modeling future distributions of monarchs, and indeed any tightly coupled plant-insect system, should incorporate the effects of climate change on host plant distributions. Finally, MaxEnt predictions of *Asclepias* and monarch distributions were remarkably consistent among general circulation models. Nearly all models predicted that the current monarch summer breeding range will become slightly less suitable for *Asclepias* and monarchs in the future. *Asclepias*, and consequently monarchs, should therefore undergo expanded northern range limits in summer months while encountering reduced habitat suitability throughout the northern migration.

## Introduction

Over the past century, climate change has altered range distributions of many species [1–3]. Numerous taxa have undergone poleward or upslope movements towards cooler temperatures and higher rainfall [4–5]. For example, lepidopterans (*i*.*e*. butterflies) have exhibited significant poleward distributional shifts due to climate warming [1, 3, 6–7]. In some cases, host switching allows more rapid northward range expansion by generalist lepidopterans than by more specialized species [8]. In contrast, if specialist lepidopterans cannot switch hosts, the rate at which they expand or shift their range will depend on range expansion of their host plant species. In cases where species are unable to shift their distributions northward or upslope due to lack of suitable habitat, *i*.*e*. a lack of host plant availability, climate change can impose severe bottlenecks or even cause extinctions [9]. Given the likely increases in greenhouse gas emissions and concomitant changes in climate, there is considerable interest in forecasting species distributions into the future to enable adequate conservation measures [10].

Monarch butterflies (*Danaus plexippus*) are a charismatic and extensively studied species, representing the quintessential migratory insect. A large population of monarchs undergoes two annual migrations. In autumn, a single generation of adult monarchs migrates south from the northern U.S. and southern Canada to overwintering sites in the mountains of central Mexico [11]. Prior to this southward migration, monarchs enter reproductive diapause [12]. Once in Mexico, monarchs remain sedentary until spring (February—March), at which point the same adults become sexually active, migrate northward, and lay the eggs of a new generation in northern Mexico and southern United States [13–14]. Monarchs continue to migrate northward in successive generations, escaping extreme temperatures and tracking the appearance of milkweed (*Asclepias spp*., hereafter *Asclepias*) [15–16]. Although several genera of plants within Apocynaceae are suitable hosts for monarch larvae [17], adult females exhibit strong ovipositional preferences for *Asclepias* and larval survival is higher on *Asclepias* compared to other species [18–20]. Monarchs occupy their breeding grounds in the midwestern and northeastern U.S. and southern Canada from May—August, where they pass through multiple generations before the southward migration in late August. Given that no single individual completes the entire migration and that there is no parental training of the migration route, it has been hypothesized that monarch migratory pathways are genetically determined [21]. Furthermore, since the northward migration occurs over multiple generations, migrating individuals must find suitable *Asclepias* larval host plants to successfully breed, ensuring that the population can complete the entire migration.

Species distributions models can provide insight into the potential impact of climate change on *Asclepias* and migratory monarchs. Previous models demonstrated that climate change might drive northward shifts in both the northern and southern range limits of monarchs [22]. However, these models only examined the ecological niche of monarch larvae. Accurate predictions of future distributions require understanding the drivers of current species distributions before using this information to model species occurrences in the future. For example, species distributions of specialist lepidopterans may be determined more by host plant availability rather than environmental effects on lepidopteran physiology *per se*, such that modeling climate change effects on lepidopteran distributions requires first modeling climate change effects on host plant distributions. Here, I report the results of a study designed to assess the effects of climate change on monarch spring migrations and their *Asclepias* host plants using maximum entropy species distribution models.

To accomplish this, I first tested hypotheses regarding physical and environmental constraints on the overall distribution of *Asclepias* and monarchs (Table 1). *Asclepias* distributions may be determined by various environmental and physical parameters, forming five hypotheses: 1) Cold temperatures limit *Asclepias*, which may be unable to withstand severe temperature swings or sustained freezes (*e*.*g*. [5]), 2) Heat limits *Asclepias*, which cannot persist beyond some critical thermal maximum, 3) *Asclepias* is precipitation limited, 4) *Aslcepias* ranges are set by geographic constraints, like elevation, slope, or land roughness, or 5) A combination of all of the above factors jointly regulates *Asclepias* distributions. It is likely that *Asclepias* is cold-limited, given its rather sharp northern range limit around the Great Lakes, but I also predicted that precipitation and warm temperatures play important roles in determining the overall distribution of *Asclepias*, such that the ‘All Variables’ hypothesis would provide the best prediction of *Asclepias* distributions.

**Table 1:** List of hypotheses for both *Asclepias* and monarchs, along with relevant predictor variables.

Hypothesis	Variables
*Asclepias*	
Cold temperatures limiting	MAT, MTCM, TAR, TS
Warm temperatures limiting	MAT, MTWQ, MTWM
Precipitation limiting	AP, PWQ, PCQ
Geographic constraints	ELEV, SLO, RGH, RUG
All Variables	All variables
	
*Monarchs*	
Habitat limited	Predicted *Asclepias* distribution
Geographic constraints	ELEV, SLO, RGH, RUG
Environmental constraints	All environmental variables
Environment and habitat	All environmental variables and predicted *Asclepias* distribution
All Variables	All variables

MAT = Mean annual temperature, MTWQ = Mean temperature of the warmest quarter, MTWM = Max temperature of the warmest month, AP = Annual Precipitation, PWQ = Precipitation of the warmest quarter, PCQ = Precipitation of the coldest quarter, MTCM = Minimum temperature of the coldest month, TAR = Temperature annual range, TS = Temperature Seasonality, ELEV = elevation, SLO = Slope, RGH = Roughness, RUG = Ruggedness.

Since *Asclepias* is a large genus comprised of both tropical, subtropical, and temperate species that may each have their own environmental constraints, I also tested the five above hypotheses on eight common North American *Asclepias* species: *A*. *curassavica*, *A*. *fascicularis*, *A*. *incarnata*, *A*. *purpurescens*, *A*. *speciosa*, *A*. *syriaca*, *A*. *tuberosa*, and *A*. *viridis*. I predicted that cold temperatures primarily control the distribution of tropical and subtropical species, such as *A*. *curassavica* and *A*. *viridis* [5]. Precipitation is likely the strongest limitation on distributions of *Asclepias* species from more arid regions, such as *A*. *speciosa* and *A*. *tuberosa*. Distributions northern *Asclepias* species are likely determined both by cold severity and warmth, such that the ‘All Variables’ model would best describe these species.

Next, I determined the factors that currently limit the distribution of eastern migratory monarchs. Flockhart et al. [16] outline three hypotheses describing constraints on monarch distributions: 1) Monarchs are limited by habitat, in particular the availability of *Asclepias* host plants [23–24], 2) Monarchs are limited purely by geographic factors, such as elevation and slope, and 3) Monarchs are limited by environmental and physiological constraints [14–15,25–26]. I here added fourth and fifth hypotheses: 4) Monarchs are limited by a combination of host plant availability plus their own innate physiological constraints, and 5) Monarchs are limited by a combination of all of the above factors. Given the tight coupling between monarchs and their *Asclepias* host plants, I predicted that the ‘Habitat’ model would provide the best fit to monarch observations.

I then used the best-fitting models to project future *Asclepias* and monarch distributions under two climate change scenarios. Finally, I examined how climate change might influence the monthly distribution of both *Asclepias* and monarchs during their northward migration from March—June. This test provides a reliable prediction of how the timing and extent of monarch northward migrations might change in the future.

## Methods

### Occurrence Data

I obtained occurrence records for adult monarchs and *Asclepias* plants from Journey North (http://www.learner.org/jnorth/), a citizen science and outreach program that tracks first appearances of both monarchs and *Asclepias* over the course of the annual spring migration [27]. The Journey North data included the GPS coordinates, accurate to the nearest postal code, for first sightings of monarchs and *Asclepias* from 2000–2011, yielding 7,717 monarch and 2,821 milkweed observations. *Asclepias* sightings from Journey North, however, were not species-specific. Therefore, these data were supplemented with species-specific records downloaded from the Global Biodiversity Information Facility (GBIF, http://www.gbif.org), which provides GPS coordinates for all observations and collections in the database. The GBIF data provided an additional 3,569 monarch observations as well as species-specific records for eight common *Asclepias* species (*A*. *curassavica* – 4,934, *A*. *fascicularis* – 1,479, *A*. *incarnata* – 899, *A*. *purpurascens* – 205, *A*. *speciosa*—991, *A*. *syriaca* – 810, *A*. *tuberosa* – 846, *A*. *virids* – 337). After coarse removal of incorrect observations (*i*.*e*. over oceans and outside North, Central, and South America), the final database included 11,277 monarch and 12,983 milkweed observations. To focus on migratory monarch populations in the eastern US, I removed monarch sightings west of the Rocky Mountains and south of the US-Mexico border. However, I retained *Asclepias* records from these locations as *Asclepias* species from these areas may expand their range into the eastern US under climate change.

### Environmental and Geogaphic Data

The current overall distributions of *Asclepias* and monarchs were modeled in relation to several bioclimatic variables in 10 arc-minute resolution based on a 50 year period (1950–2000) downloaded from the WorldClim website (http://www.worldclim.org) [28]. To model geographic constraints, I obtained a raster of global elevation data and calculated slope, roughness (the absolute value of the difference between minimum and maximum of a cell and its eight neighbors), and ruggedness (mean absolute value of the difference between a cell and each of its eight neighbors). To test specific hypotheses regarding *Asclepias* distributions outlined above, I isolated several variables pertaining to each hypothesis (Table 1).

I also combined variables to test hypotheses regarding monarch distributions (Table 1). Flockhart et al. [16] used NDVI and percent vegetation cover derived from MODIS images to model habitat availability for monarchs. However, I did not include those variables here, as projections of NDVI and percent habitat under climate change do not yet exist and could not be included in model projections of future distributions should they be important, as was likely. Instead, I used projections of overall *Asclepias* distributions derived from the MaxEnt models of *Asclepias* to estimate habitat availability (Table 1). *Asclepias* distribution is likely related to NDVI and percent habitat availability, such that this variable accurately portrays monarch habitat availability in lieu of other metrics for which climate change projections are unavailable.

For monthly distributions, only monthly mean temperature, monthly maximum temperature, and monthly precipitation were available under climate change scenarios. Therefore, I use all three of these variables to model monthly *Asclepias* distributions during the northward migration (March—June). I used these three variables plus the monthly *Asclepias* distribution to model monthly monarch distributions.

### Climate Change Projections

Predictions of the same bioclimatic variables for the 2080s were downloaded from the Climate Change, Agriculture, and Food Security website (http://www.ccafs-climate.org). Climate predictions were based on four general circulation models (GCMs: BCCR, IPSLCM4, MIROC, and NCAR) under two emissions scenarios for each GCM: moderate (B1) and severe (A2). The B1 scenario assumes that greenhouse gas emissions rise slowly until 2050 and decline thereafter, resulting in a moderate 1°–3° C increase in mean global atmospheric temperatures [29]. The A2 scenario assumes greenhouse gas emissions rise steadily through 2100, resulting in a more severe 2°–6° C increase in mean global atmospheric temperatures [29]. I used the most up to date predictions available from the CCAFS website.

In addition to the summary bioclimatic variables, I also downloaded current and future estimates of monthly mean temperature, monthly maximum temperature, and monthly precipitation. Monthly climate data were at the same resolution as the bioclimatic variables and future predictions were based on the same GCMs and emissions scenarios as above.

### Species Distribution Modeling

As occurrence data for monarchs and *Asclepias* were presence-only, I used maximum entropy methods (MaxEnt) to estimate species distributions of both *Asclepias* and monarchs [30]. MaxEnt uses Bayes’ rule to estimate the probability of species occurring in each raster cell given the underlying environmental characteristics. To model the probability of occurrence at a site based on environmental characteristics, Bayes’ rule implies that one need know only the probability of environmental characteristics given an observation (*i*.*e*. the distribution of the environmental variables at each observed point), the overall distribution of environmental variables, and the overall probability of species occurrences, which is a constant [31]. MaxEnt calculates the relative occurrence at each site (*e*.*g*. log odds ratio of occurring in site) by maximizing the environmental similarity between suitable sites and the background environment, while constraining the predictions to have the same environmental characteristics as the observations [32]. That is, MaxEnt attempts to make suitable sites as similar as possible to the background distribution, while constraining them to have the same mean as the observations (*i*.*e*. the mean temperature of the suitable predictions should have the same mean temperature as the observations). The relative occurrence predictions are converted to probability of occurrence using a variant of the logistic transformation, assuming that the probability of presence at a location of average suitability is 0.5 [31–32]. This assumption means that a species is as likely to be present as it is absent in a location of ‘average’ suitability. Elith et al. [31] and Merow et al. [32] provide a more comprehensive overview of the mechanics underlying MaxEnt.

**Fig 1:**
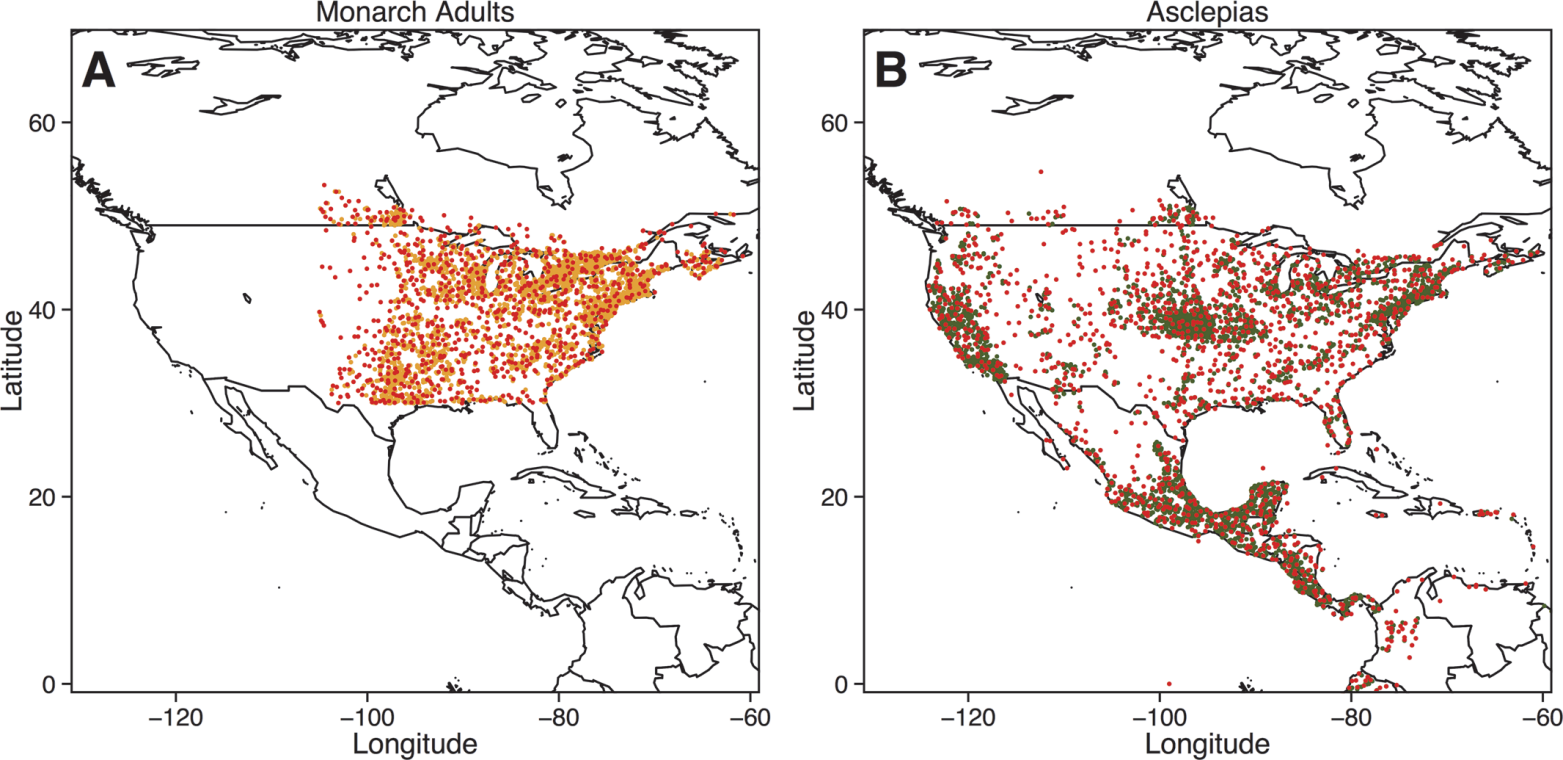
Observation records for adult monarchs and *Asclepias* gathered from both Journey North and GBIF. Note the extreme spatial bias around urban population centers in both datasets. The red points depict the spatially filtered observations used in MaxEnt models.

Monarch and *Asclepias* data were analyzed separately following similar procedures. First, environmental data were clipped to North America, Central America, and northern South America, including areas just outside of the range of *Asclepias*. At a 10 arc-minute resolution, this yielded just over 120,000 raster cells for prediction. The Journey North data showed considerable clumping around major cities in the midwest, southwest, and northeast United States (Fig. 1). Such spatial bias can artificially inflate the accuracy of SDM predictions and restrict the range of environments predicted as suitable [33]. Despite its simplicity, spatial filtering (*i*.*e*. thinning observations within larger grid cells) counters spatial bias more effectively and consistently than numerous other techniques [33–34]. For spatial filtering, I created a grid covering the sampling area consisting of cells with a resolution 420% larger than the original 10 arc-minute resolution (~40 arc-minute resolution). Cells were sampled in a checkerboard pattern, choosing one observation at random within designated cells while cells without any observations remained empty. This reduced the number of observations to 878 for monarchs and 1,444 for *Asclepias* (Fig. 1). This thinning resolution provided a reasonable trade-off between bias reduction and sample size (results from other spatial filtering schemes available in S1 Appendix).

After spatial filtering, observations were randomly split into two subsets. One data subset was used as model training data for MaxEnt. The remaining group was then used for model validation (~700 for both training and test data for *Asclepias*, ~450 in each group for monarchs). To test each hypothesis, I ran models pertaining to each hypothesis (Table 1) and used AICc for MaxEnt models, as described by Warren and Seifert [35], to choose the best-fitting model. To measure goodness-of-fit, I calculated the area-under-the-curve (AUC) statistic that provides an estimate for the accuracy of predictions, with 0 being no predictive accuracy and 1 being perfect predictive accuracy. A score of 0.5 indicates that the model performs no better than random.

The best-fitting MaxEnt models were then used to project *Asclepias* and monarch distributions into the future based on the changes in climate predicted under each of the four GCMs and for each of the two emissions climate scenario (eight total future models per species). To determine overall species’ distributions, occurrence data from all years and all months were pooled and input to the model simultaneously. To determine species-specific responses to climate change, I repeated the same procedure described above for the GBIF data on each of the eight *Asclepias* species separately (Journey North data could not be used as it was not identified to species). I used spatial filtering on the same sized grid (~40 arc-minute resolution) to account for any spatial bias in observations. Although filtering diminished the number of points available for distribution modeling (*A*. *curassavica*—327, *A*. *fascicularis* – 154, *A*. *incarnata* – 275, *A*. *purpurascens* – 58, *A*. *speciosa* – 313, *A*. *syriaca* – 157, *A*. *tuberosa* – 238, *A*. *viridis* – 83, S1 Fig.), it helps avoid reporting spurious correlations with environmental parameters that might result from biased record collections (S1 Fig.) [33]. Such small sample sizes may not capture the full range of environmental conditions occupied by these species, as such these analyses should be considered exploratory examinations of potential climate change effects of specific *Asclepias* species.

Climate change often alters plant phenology, wherein plants emerge earlier or later in the year [36]. Climate change may therefore alter the timing of *Asclepias* emergence and monarch northward migrations. To determine how climate change might affect the monthly appearance of *Asclepias* and monarchs, I isolated the first *Asclepias* sightings for each month from the Journey North data and fit a MaxEnt model using monthly mean temperature, maximum temperature, and precipitation (occurrence data were first prepared as described above). In these models, points lacked obvious spatial bias, and some months had few observations. Therefore, I did not use spatial filtering to subsample observations. Results from all four GCMs were averaged by averaging predicted probabilities of occurrence for each cell from each of the models to yield a single ensemble estimate of the effects of climate change on *Asclepias* phenology. These same analyses were repeated for first sightings of monarch adults, except models also included the predicted first sightings of *Asclepias* for each climate change scenario.

All statistical analyses were carried out in R v3.1.2 and the ‘*raster’* and ‘*dismo*’ packages using MaxEnt software [30,37]. All predictors were standardized prior to analyses. All data are publicly available at Journey North or GBIF.

## Results

### Current and Future Asclepias Distributions

**Fig 2:**
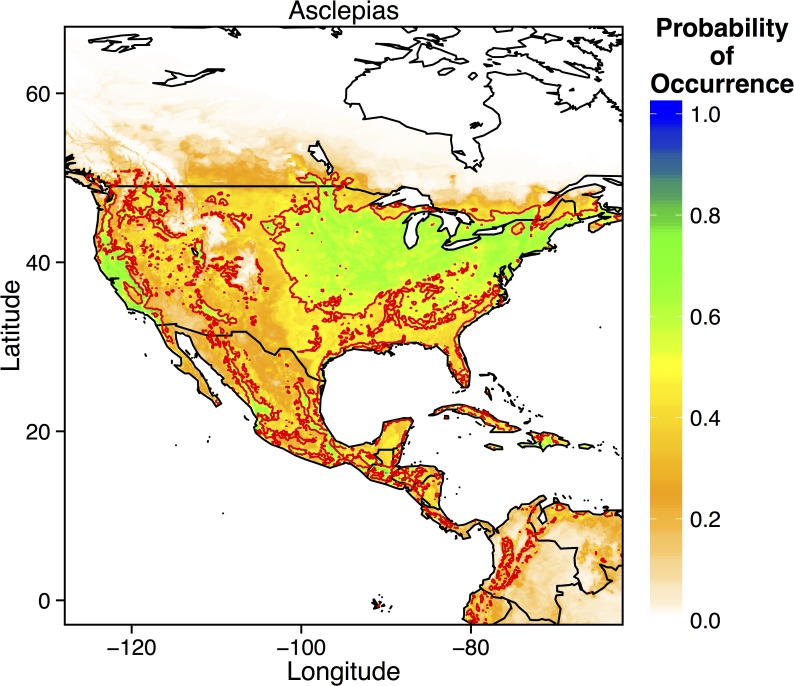
Prediction from the best-fitting MaxEnt model for the probability of occurrence of the overall *Asclepias* distribution across North, Central, and parts of South America. Thick red lines denote the 0.5 probability contour, such that areas inside the contour have a ≥ 0.5 probability of containing *Asclepias*.

The overall *Asclepias* distribution was best explained by a combination of all environmental and geographic variables (Table 2). Indeed, no other hypothesis had a reasonable level of support, such that ΔAICc of the next best hypothesis was 1782.34. Moreover, the ‘All Variables’ hypothesis provided the best goodness-of-fit (AUC = 0.805). In this model, mean annual temperature explained over half of the variance, as *Asclepias* only occurred in areas where mean temperatures were above 0° but below 30° C. Mean temperature of the warmest quarter and minimum temperature of the coldest month also explain ~ 20% of the variance each. Thus, while all predictors were important in modeling the distribution of *Asclepias*, it appears that temperature provides the primary constraint on *Asclepias* distributions. MaxEnt therefore predicted that *Asclepias* should occur most frequently in central and eastern US, along the west coast of the US, and throughout much of Mexico and Central America (Fig. 2).

**Table 2:** Results of AICc model selection for overall *Aslcepias* distribution and each species.

Hypothesis	n	AICc	ΔAICc	AUC
*Overall Asclepias*				
**All**	**129**	**28633.89**	**0**	**0.805**
Geographic	58	30416.22	1782.34	0.589
Cold	66	31074.12	2440.24	0.760
Warm	42	31198.06	2564.17	0.749
Precipitation	63	32137.08	3503.20	0.704
*A*. *curassavica*				
**All**	**81**	**5711.23**	**0**	**0.918**
Geographic	63	6381.29	670.06	0.842
Cold	46	7034.69	1323.46	0.923
Warm	44	7239.18	1527.94	0.888
Precipitation	53	7680.42	1969.18	0.830
*A*. *fascicularis*				
**All**	**67**	**2748.98**	**0**	**0.981**
Cold	20	2807.28	58.30	0.951
Warm	17	2900.98	152.01	0.950
Precipitation	46	2966.72	217.74	0.936
Geographic	25	3100.38	351.40	0.836
*A*. *incarnata*				
**All**	**96**	**5473.78**	**0**	**0.908**
Warm	35	5559.95	86.17	0.903
Cold	40	5580.12	106.34	0.900
Precipitation	41	5950.71	476.94	0.827
Geographic	38	6084.38	610.61	0.695
*A*. *purpurascens*				
**Cold**	**12**	**1119.10**	**0**	**0.967**
Warm	10	1123.09	3.99	0.963
All	34	1165.03	45.93	0.983
Precipitation	16	1191.92	72.82	0.932
Geographic	28	1371.67	252.57	0.863
*A*. *speciosa*				
**Cold**	**48**	**6297.64**	**0**	**0.904**
**All**	**102**	**6297.69**	**0.05**	**0.919**
Warm	39	6345.62	47.98	0.897
Precipitation	53	6684.41	386.77	0.849
Geographic	46	6866.18	568.54	0.747
*A*. *syriaca*				
**Warm**	**14**	**2929.61**	**0**	**0.941**
All	52	2953.44	23.83	0.948
Cold	10	2955.62	26.01	0.927
Precipitation	33	3229.32	299.70	0.876
Geographic	25	3379.25	449.63	0.747
*A*. *tuberosa*				
**All**	**94**	**4894.67**	**0**	**0.914**
Warm	32	4936.86	42.19	0.888
Cold	42	4963.61	68.94	0.886
Precipitation	32	5309.36	414.69	0.779
Geographic	58	5509.82	615.14	0.659
*A*. *viridis*				
**Warm**	**17**	**1511.33**	**0**	**0.966**
Cold	15	1532.98	21.65	0.954
All	47	1592.19	80.86	0.967
Precipitation	25	1764.22	252.89	0.800
Geographic	46	1921.07	409.74	0.783

Hypotheses are as described in Table 1. *n* gives the number of parameters in each model, which was calculated as the number of non-zero λ values from each MaxEnt model.

Species distribution models of individual *Asclepias* species provided a much better goodness-of-fit (AUC > 0.9 for all species), although some of the high predictive success was probably due to low sample size (*e*.*g*. *A*. *purpurascens*). However, species showed considerable variability in the best predictors of current distributions. For example, the ‘All Variables’ model best explained the distributions of *A*. *curassavica*, *A*. *fasciularis*, *A*. *incarnata*, and *A*. *tuberosa* (Table 2). *Asclepias purpurascens* and *A*. *speciosa* appear to be cold-limited, although the ‘All Variables’ model explained the distribution of *A*. *speciosa* equally well (Table 2). Finally, *A*. *syriaca* and *A*. *viridis* appear to be primarily limited by warm temperatures (Table 2). Likewise, there was considerably variability in the predictors that contributed most to the variance species distributions. Temperature seasonality and minimum temperature of the coldest month explained most of the variance in *A*. *curassavica* observations, while *A*. *fascicularis* was limited by precipitation in the warmest quarter, terrain roughness, and temperature seasonality (S2 Fig.). Temperature seasonality and mean annual temperature also regulated the distribution of *A*. *incarnata* and *A*. *purpurascens*, although warmest quarter temperatures and precipitation also strongly influenced *A*. *incarnata* (S2 Fig.). Distributions of *A*. *speciosa*, *A*. *syriaca*, and *A*. *viridis* were primarily influenced by mean annual temperature (S2 Fig.). *Asclepias tuberosa* appears to be influenced by a range of temperature and precipitation related variables (S2 Fig.).

**Fig 3:**
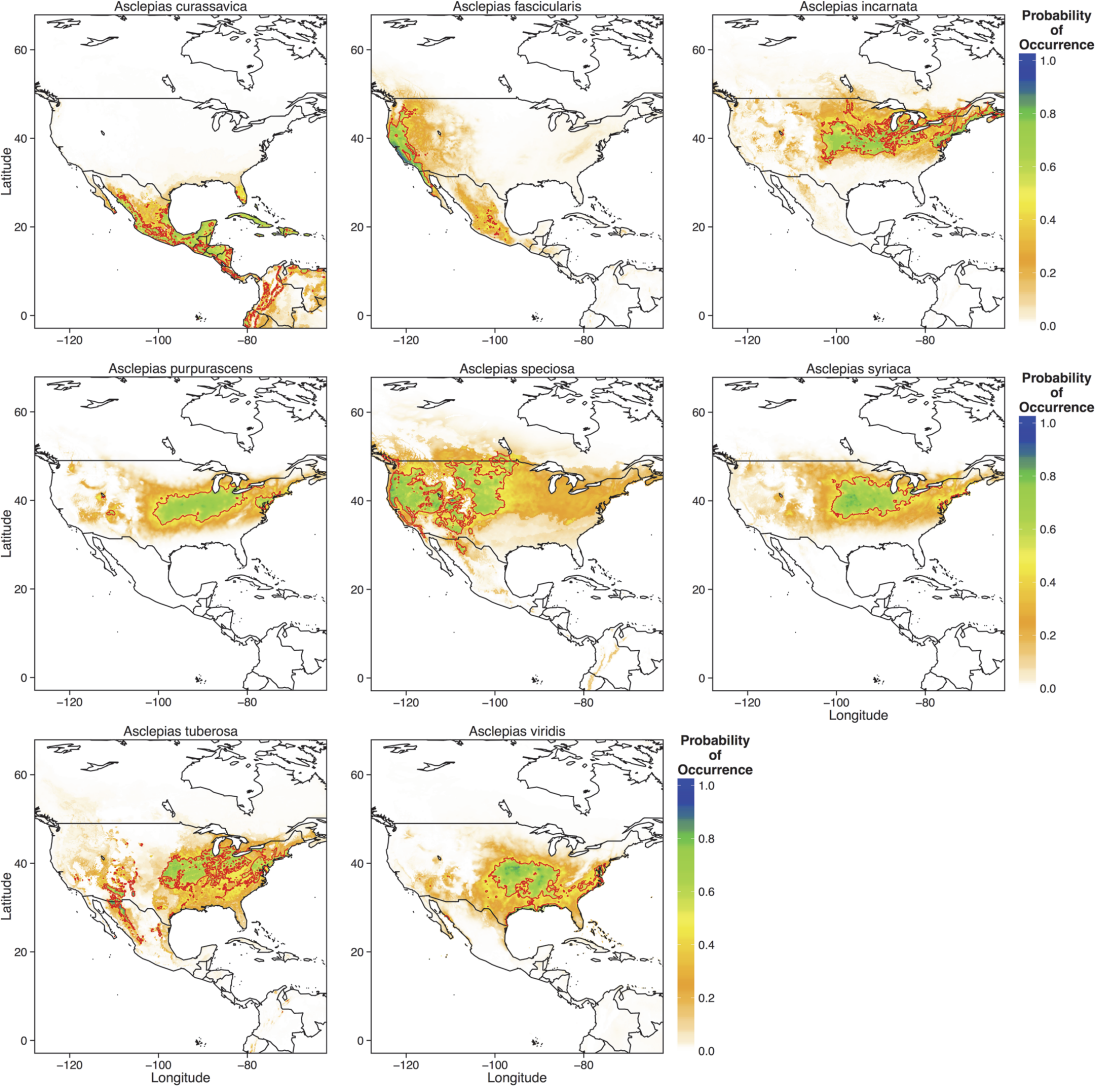
Predictions for the probability of occurrence of each of the eight *Asclepias* species across North, Central, and parts of South America. Thick red lines denote the 0.5 probability contour, such that areas inside the contour have a ≥ 0.5 probability of containing *Asclepias* or the individual species.

MaxEnt predicted that *A*. *curassavica* should currently occur along the coasts of Mexico and throughout Central America and the Caribbean (Fig. 3). *Asclepias fascicularis* had a predicted range relegated to small parts of the west coast of the US, whereas *A*. *speciosa* was predicted to occur through most of the western United States (Fig. 3). *Asclepias incarnata*, *A*. *purpurascens*, and *A*. *syriaca* were predicted to occur throughout the midwestern and northeastern United States (Fig. 3). *Asclepias tuberosa* had the largest predicted range, with high probabilities of occurrence in the southwestern, midwestern, and northeastern United States (Fig. 3). These predicted distributions are nearly identical to those that have been historically reported for these species [38].

**Fig 4:**
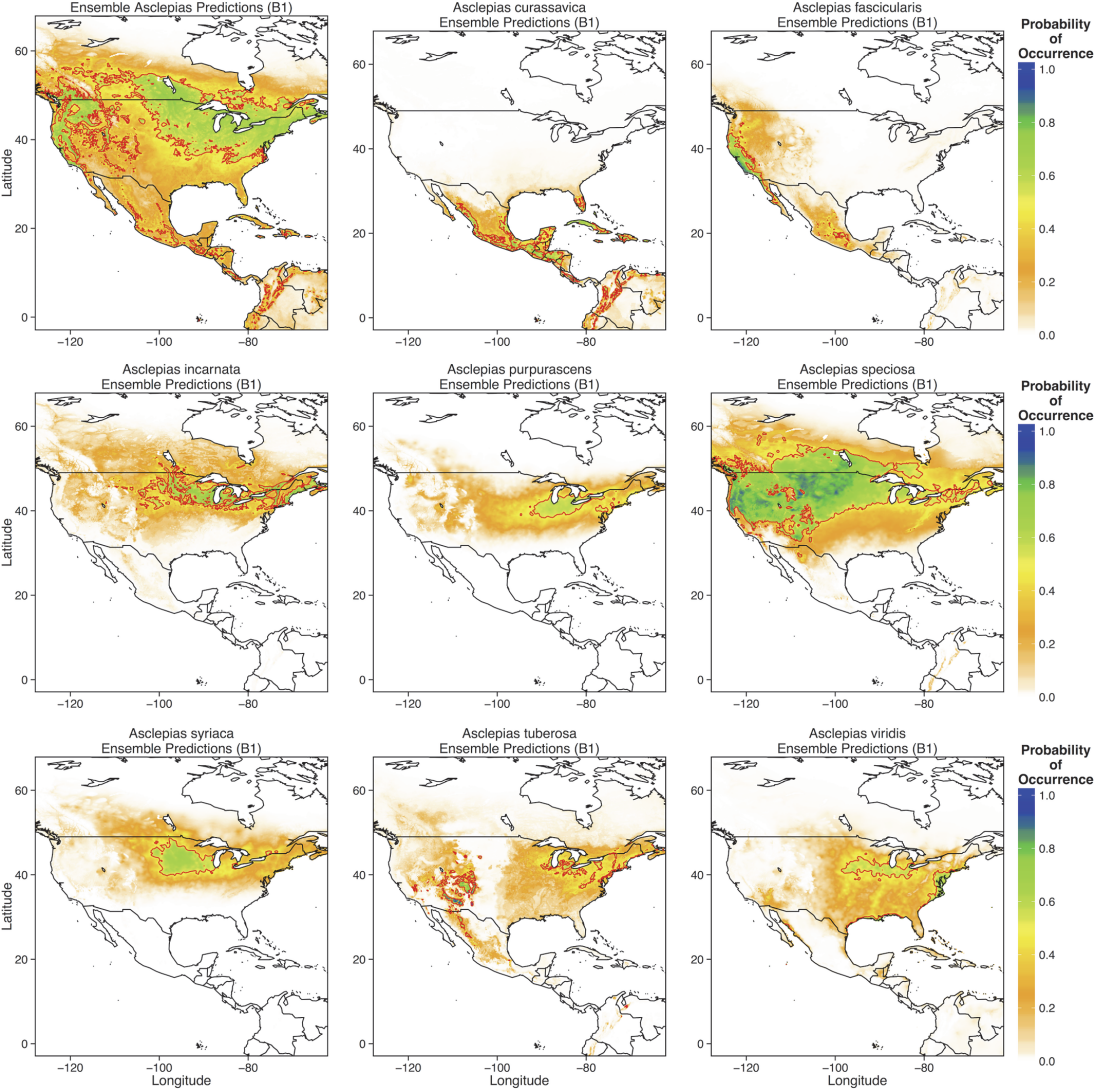
Ensemble predictions of overall *Asclepias* distribution and each species for all four GCMs under the moderate climate change scenario (B1). Ensemble predictions were created by averaging model output from all four GCM predictions. The thick red line denotes the 0.5 probability contour, such that areas inside the contour have a ≥ 0.5 probability of containing *Asclepias* or the individual species.

Given that the overall *Asclepias* distribution and that of many individual species was constrained primarily by temperature, climate change should have significant effects on the potential distribution of *Asclepias spp*. Indeed, under moderate climate change (scenario B1), the overall distribution of *Asclepias* was projected to expand northward into much of Canada, including much of Manitoba and Ontario (Fig. 4). Additionally, much of the southern United States became less suitable for *Asclepias*. Individually, *Asclepias* species exhibited substantial variation in their responses to moderate climate change. Suitable areas for *A*. *curassavica*, *A*. *tuberosa*, and *A*. *viridis* (probability of occurrence > 0.5) became significantly reduced under moderate climate change scenarios (Fig. 4). In contrast, the northward ranges of *A*. *incarnate*, *A*. *speciosa*, *A*. *syriaca*, and *A*. *viridis* expanded substantially, with *A*. *speciosa* predicted to occur in much of southern Canada (Fig. 4).

**Fig 5:**
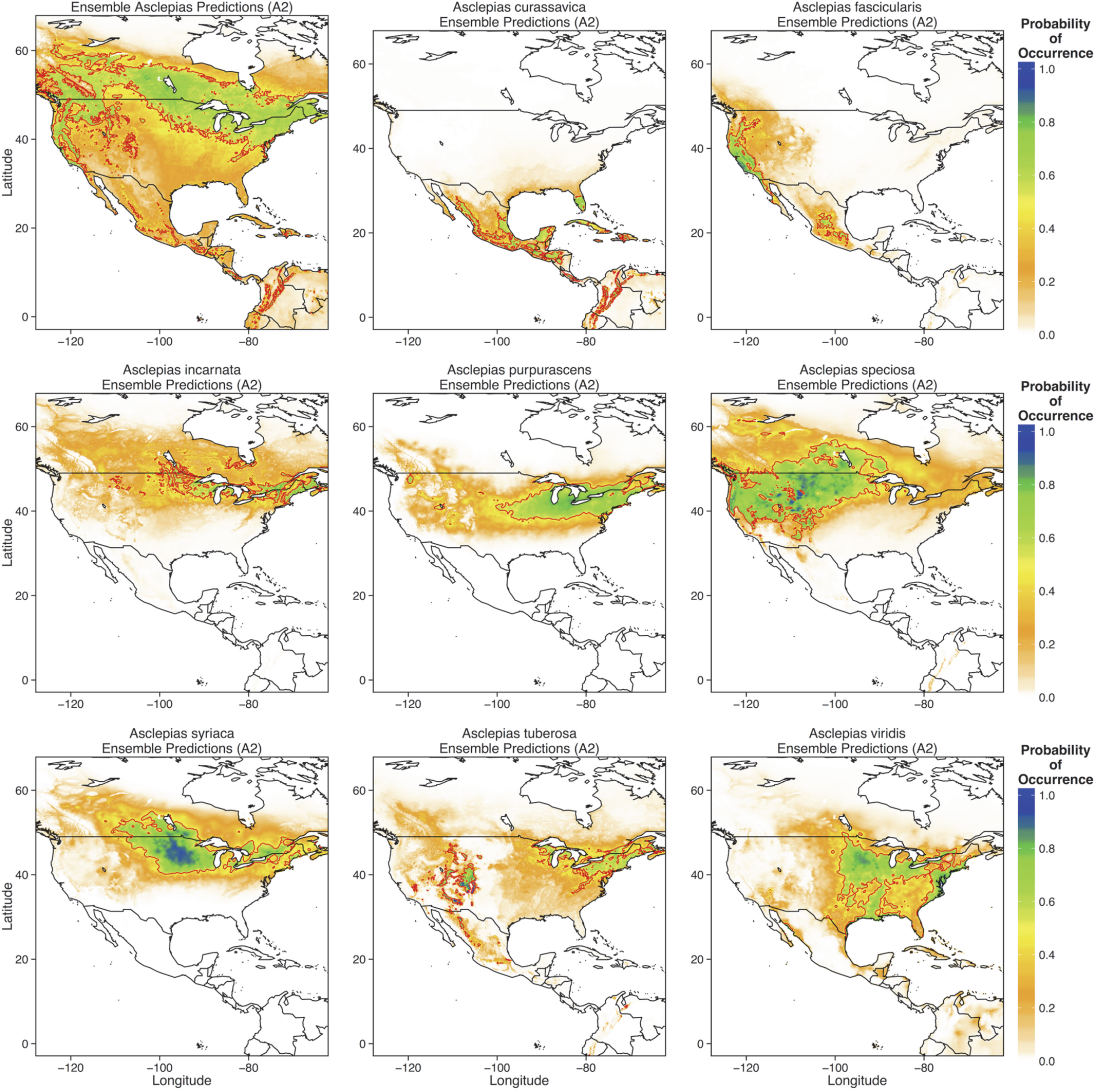
Ensemble predictions of overall *Asclepias* distribution and each species for all four GCMs under the severe climate change scenario (A2). Ensemble predictions were created by averaging model output from all four GCM predictions. The thick red line denotes the 0.5 probability contour, such that areas inside the contour have a ≥ 0.5 probability of containing *Asclepias* or the individual species.

MaxEnt predicted that severe climate change (scenario A2) yields similar changes to the overall distribution of milkweeds, but less severe reductions in the extent of suitable area for most milkweed species (Fig. 5). Overall, much of Canada became suitable habitat for *Asclepias* species, and the northward range expansion of *A*. *incarnata*, *A*. *speciosa*, *A*. *syriaca*, and *A*. *viridis* was more pronounced than under moderate climate change scenarios (Fig. 5). Interestingly, *A*. *viridis* was predicted to occupy most of the eastern United States, whereas *A*. *incarnate*, *A*. *syriaca*, and *A*. *tuberosa* should become restricted to the northern United States and southern Canada (Fig. 5)

### Current and Future Monarch Distributions

**Fig 6:**
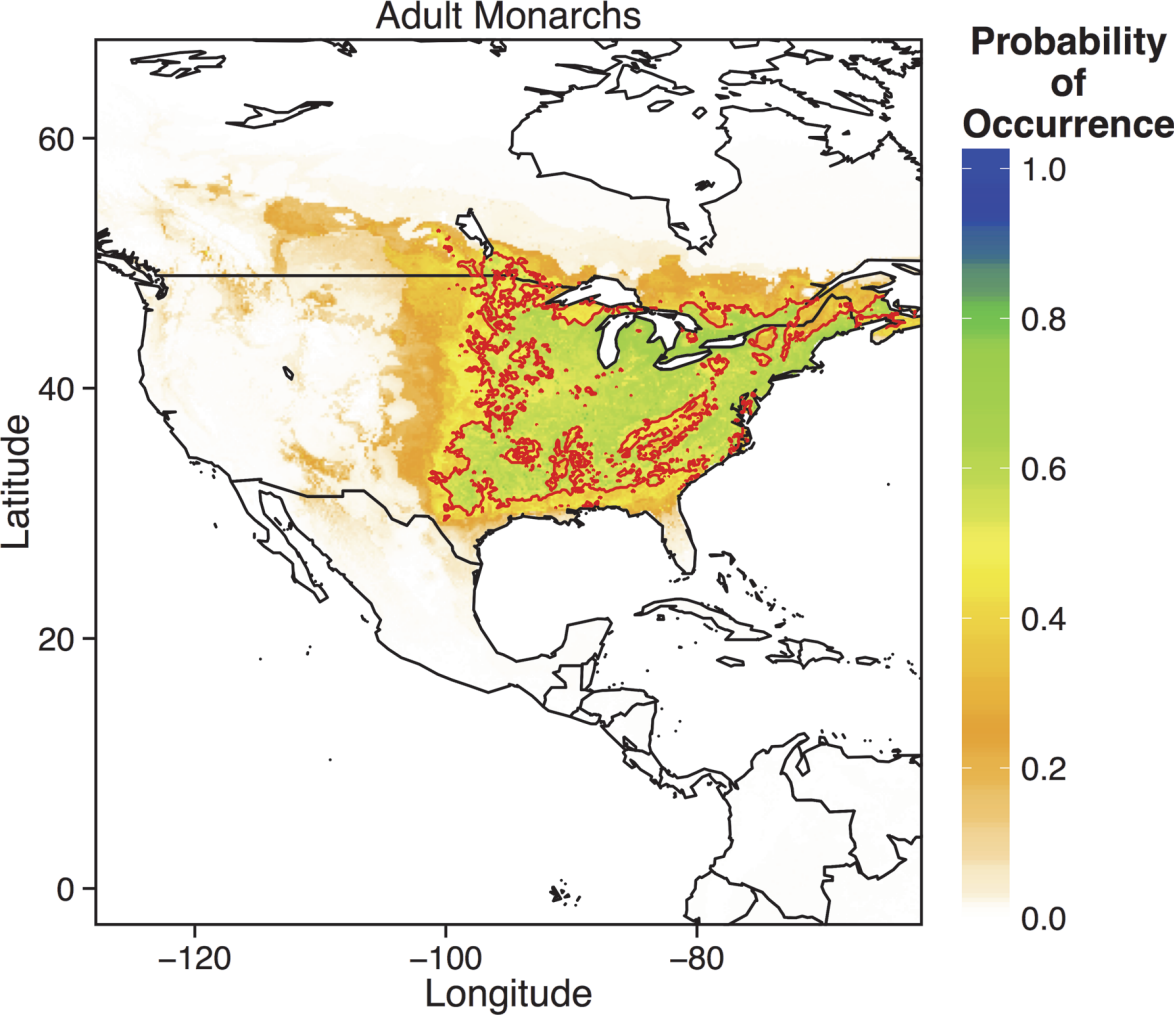
Prediction from the best-fitting MaxEnt model for the probability of occurrence of adult monarchs (*Danaus plexippus*) throughout eastern North America. Thick red lines denote the 0.5 probability contour, such that areas inside the contour have a ≥ 0.5 probability of containing monarchs.

Monarch distributions were best explained by a combination of habitat availability and environmental constraints, such that the ‘Environment + Habitat’ model fit the data well (AUC = 0.888, Table 3). The ‘All Variables’ model performed second best, but relatively large AICc values compared to the best-fitting model suggest that this model is unlikely to be the best model (ΔAICc = 32.12) (Table 3). In particular, the predicted *Asclepias* distribution explained 35% of the variance in monarch observations. Temperature seasonality, precipitation of the warmest quarter, mean annual temperature, and maximum temperature of the warmest month were also important predictors of monarch observations. MaxEnt predictions of eastern migratory adult distributions accurately depicted the known distribution (Fig. 6). Monarch adults were predicted to occur through the central and midwestern US, along the east coast (except for the Appalachian Mountains), and throughout the northeastern US (Fig. 6). The northern range limit of eastern migratory adults lies around the Great Lakes region.

**Table 3:** Results of AICc model selection for adult monarchs.

Hypothesis	n	AICc	ΔAICc	AUC
**Environment and habitat**	**79**	**16355.06**	**0**	**0.888**
All	93	16387.18	32.12	0.892
Habitat	24	17102.21	747.15	0.831
Environment	73	17766.22	1411.16	0.896
Geographic	58	18701.64	2346.58	0.697

Hypotheses are as described in Table 1. *n* gives the number of parameters in each model, which was calculated as the number of non-zero λ values from each MaxEnt model.

**Fig 7:**
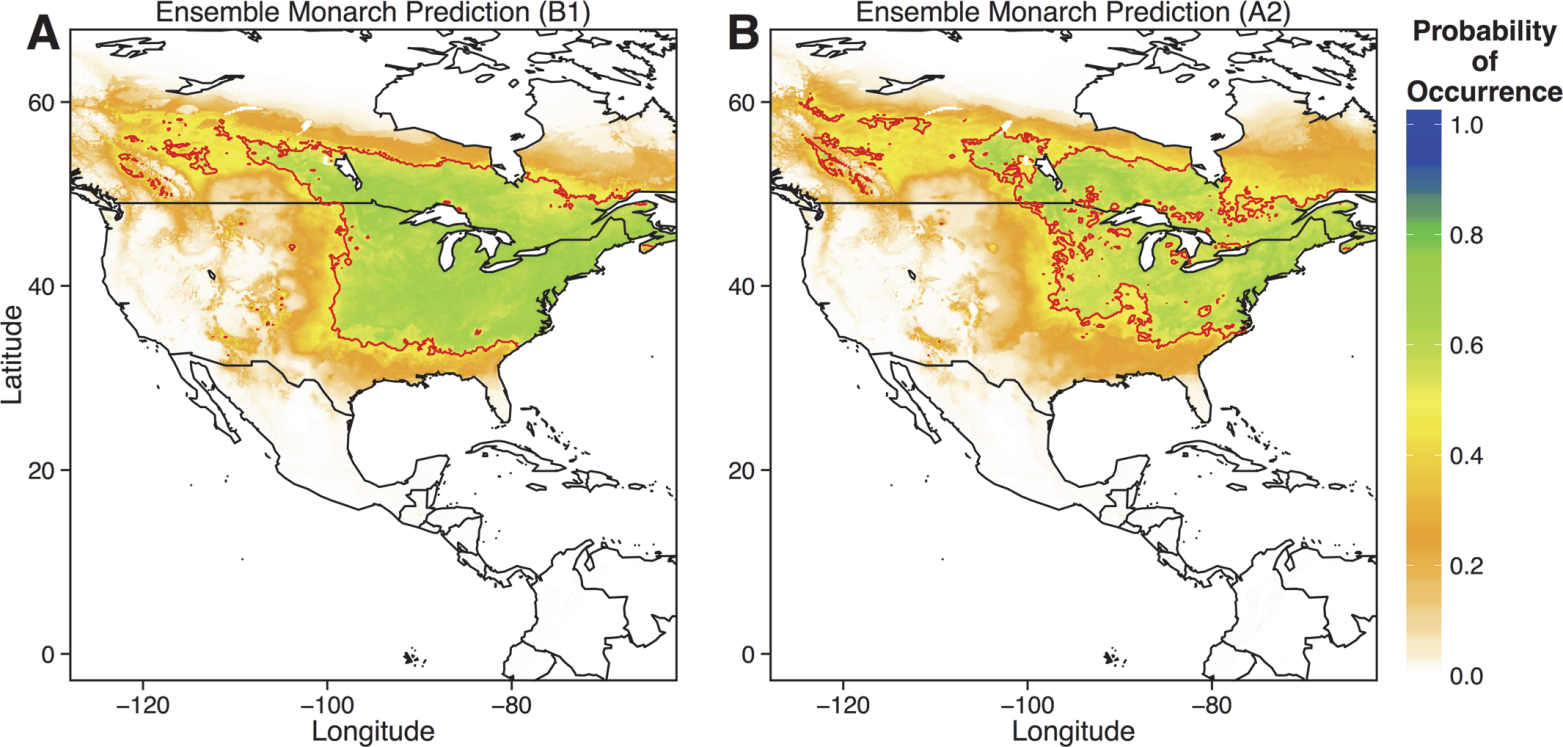
Ensemble predictions of monarch distribution under moderate (B1) and severe (A2) climate change scenarios. Ensemble predictions were created by averaging model output from all four GCM predictions. Predicted occurrence of *Asclepias* under each climate change scenario was used as a predictor in each model. The thick red line denotes the 0.5 probability contour, such that areas inside the contour have a ≥ 0.5 probability of containing monarchs.

Given that monarch distributions are therefore controlled by physiological temperature constraints and host-plant availability, which itself is sensitive to climate change, climate change should have a considerable influence on monarch distributions. In fact, both moderate and severe climate change scenarios yielded similar predictions (Fig. 7). Both the northern and southern range limits of eastern migratory monarchs shifted northward. The northern range limit extended throughout eastern Canada, while the southern range limit resided in the central US, rather than along the Gulf of Mexico (Fig. 7). These results are similar to those of earlier models by Batalden et al. (2007).

### Climate Change Effects on Monthly Distributions of *Asclepias*

**Fig 8:**
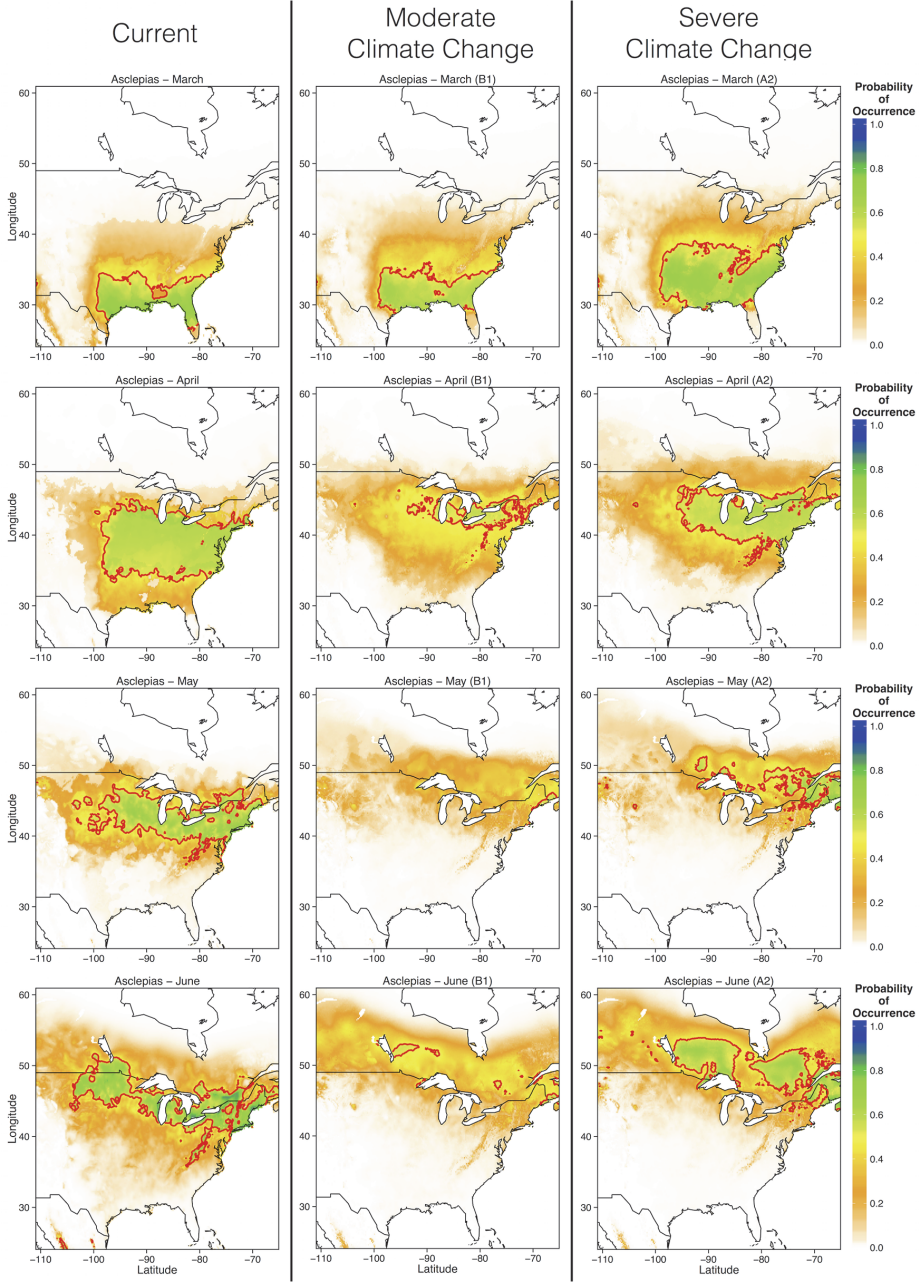
Ensemble projections of future *Asclepias* distributions during each of the spring months (March—June) under moderate and severe climate change scenarios. Ensemble projections were generated by averaging MaxEnt output from each of the four GCM predictions.

**Fig 9:**
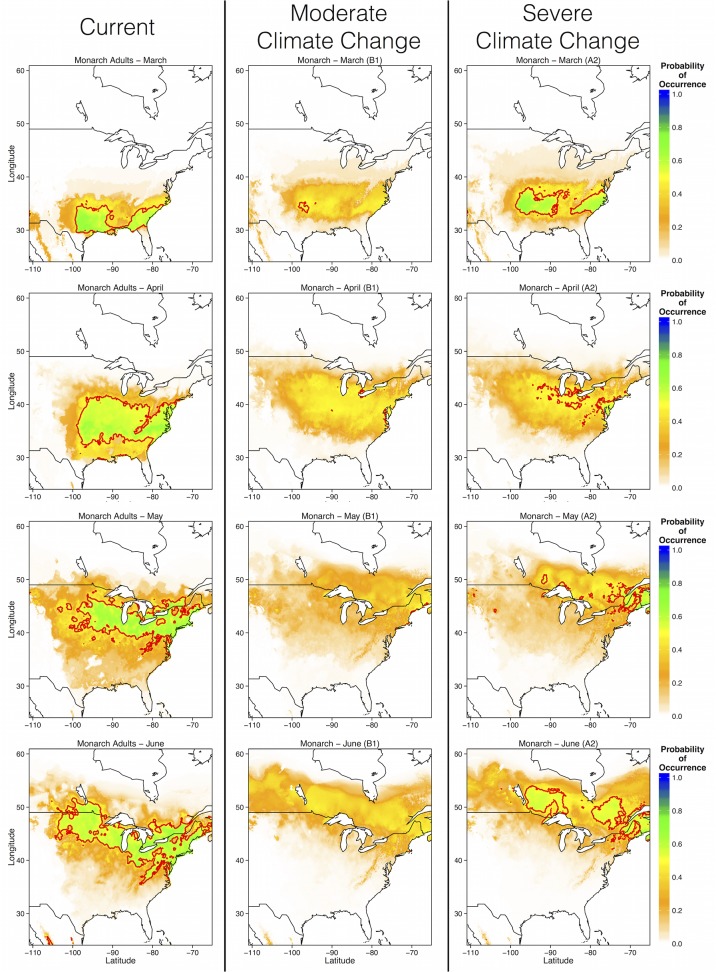
Ensemble projections of future monarch distributions during each of the spring months (March—June) under moderate and severe climate change scenarios. Ensemble projections were generated by averaging MaxEnt output from each of the four GCM predictions.

First sightings of *Asclepias* occurred progressively further north over the course of spring. In March, cold temperatures restrict *Asclepias* to warmer southern and southeastern U.S. In April, first sightings of *Asclepias* occured throughout the eastern U.S. However, first sightings of *Asclepias* in the Great Lakes region, the monarch’s summer breeding grounds of eastern populations, did not occur until May and June (Fig. 8). Under the moderate emissions scenario, suitable area for first sightings of *Asclepias* was reduced in all months (Fig. 8). Also, *Asclepias* did not cease expanding northward at the Great Lakes in May, as it currently does, but continued to move poleward through June (Fig. 8). Severe climate change also reduced the amount of area suitable for first *Asclepias* sightings, but not as severely as moderate climate change. Indeed, the area suitable for first *Asclepias* emergence in March occupied much of the southern U.S. and Atlantic coast under the more severe emissions scenario. In June, the northern limit of first *Asclepias* emergence was further north than currently predicted, as with moderate emissions, but the environmental conditions were more suitable for *Asclepias* than under moderate emissions scenarios (Fig. 8). Monarchs exhibited similar phenological patterns, with reduced probability of first sightings throughout the eastern range, and a greatly expanded northward range limit in May and June (Fig. 9). Thus, monarchs may extend their northward migration beyond the Great Lakes region into much of southern Canada, following the appearance of milkweed.

## Discussion

As climate change progresses, many species escape unfavorable temperatures or colonize previously intolerable habitats via northward range expansion. Lepidopterans, especially butterflies, seem especially adept at capitalizing on newly available habitat at their northern limits [1,6–7]. Given their ecological importance, both in natural communities and as pests in agricultural settings, increased attention has been given to predicting lepidopteran distributions under future climates (*e*.*g*. [39]). Yet many lepidopteran species specialize on one or a few host plant species and the ecological niche of their host plant(s) govern their geographic range as strongly as environmental factors [39]. In contrast, monarchs can utilize a large number of different hosts, albeit most within the genus *Asclepias*. My results demonstrate that the modeled ecological niches of monarchs are most accurate when incorporating the predicted distribution of their *Asclepias* host plants alongside important environmental predictors. Both monarchs and *Asclepias* distributions appear to be constrained by precipitation and temperature, and the distribution of *Asclepias* accounts for much of the variability in monarch observations. Given this strong interspecific dependence, projecting *Asclepias* distributions under climate change scenarios is crucial to understanding how climate change will alter monarch distributions.

Numerous studies have used ecological niche modeling to predict changes in species distributions wrought by climate change. In many cases, these models contain implicit assumptions about lability of species interactions under future climatic conditions [40]. Using only environmental variables to forecast lepidopteran distributions in future climates implicitly assumes that the host plant will shift in similar ways (*i*.*e*. for specialists) or that biological interactions are sufficiently plastic that the lepidopteran is not constrained by the distribution of any specific host plant (*i*.*e*. for generalists). Generalist lepidopterans exhibit substantial range expansions due to warming temperatures, in part because they can shift host plants [6–8]. However, some species, like monarchs, are largely dependent upon a single genera or species of host plants. In such cases, it is difficult to disentangle the climatic niches of the host plant and the herbivore [41]. This may be why lepidopterans as a group exhibit such highly variable range expansions; many have not shifted north or south at all despite considerable warming [3]. I show that monarch and *Asclepias* ecological niches overlap considerably and were difficult to distinguish (Table 3); the predicted *Asclepias* distribution explained ~ 35% of the variation in monarch observations. I therefore suggest that modeling climate change effects on *Asclepias* is necessary, although not sufficient, to accurately represent potential climate change effects on monarch distributions.

Currently, the northern range limit of monarchs lies slightly north of the U.S.—Canada border, just above the Great Lakes [13,16]. My ecological niche models of current monarch and *Asclepias* distributions match these observations, predicting a northern range limit just north of the Great Lakes (Fig. 2 and Fig. 6). MaxEnt models of specific *Asclepias* species accurately reconstructed the ranges historically reported for many of these species (Fig. 3, [38]). The MaxEnt model of monarch occurrences including *Asclepias* distribution as a predictor provided nearly the same estimate of monarch distribution as previous models [16]. Indeed, summer monarch breeding grounds of the northern Midwest and northeast U.S. are highly suitable for *Asclepias* (Fig. 2 and Fig. 8). Under both moderate and severe emissions scenarios, much of *Asclepias’* current range is predicted to become slightly less suitable, although the probability of *Asclepias* occurring in these areas remains high (Fig. 4 and Fig. 5). Under moderate climate change, the northern range limit of *Asclepias* may extend slightly into southern Manitoba, Ontario, and Quebec (Fig. 4). Under severe climate change, the northern range limit expands even further and much of Manitoba, Ontario, and Quebec become suitable for *Asclepias*. In part, this is because both climate scenarios predict warmer average temperatures throughout much of Canada (5–7° C increase), severely decreased rainfall during the summer throughout the midwestern United States (40–60 mm decrease), and increased average summer temperatures through much of Canada (4–7° C increase). The northward expansion of *Asclepias* into Canada led to MaxEnt projecting a substantial northward range expansion of monarchs under moderate and severe climate change (Fig. 7).

However, not all *Asclepias* species responded similarly to climate change, although most showed an increased northern range limit (Fig. 3). The distribution of *A*. *curassavica* was primarily determined by temperature seasonality and minimum temperature of the coldest month. As *A*. *curassavica* is primarily a tropical species (S1 Fig.), this fits with the long-standing hypothesis that tropical species are adapted to warm climates with little variability [42]. Accordingly, tropical species often show reduced thermal tolerance ranges compared to temperate species and should therefore be affected more strongly by increased temperature seasonality [43]. Additionally, tropical species often show sensitivity to extreme cold events, such as the minimum temperature of the coldest month or number of days below a critical temperature threshold [5]. Although minimum cold temperature is expected to increase in Mexico and Central America, a concomitant increase in temperature seasonality appears to restrict the range of *A*. *curassavica* under future climate scenarios. In contrast, *A*. *incarnata* and *A*. *syriaca* are primarily temperature-limited and will likely see increased range sizes in the future as more of Canada experiences temperatures suitable for these species.

The differing responses of *Asclepias* species to climate change could potentially impact monarch populations. In laboratory trials, adult females exhibited strong ovipositional preferences for some *Asclepias* species over others, for example preferring *A*. *incarnata* to *A*. *syriaca*, presumably due to differing cardenolide concentrations [44]. Further, larval growth and survival is often higher on *A*. *incarnata* and *A*. *curassavica* compared to the more common *A*. *syriaca* [44–45]. My MaxEnt models predict that *A*. *syriaca* and *A*. *incarnata* should become restricted to more northern parts of North America, whereas other host species such as *A*. *speciosa* and *A*. *viridis* should become widespread throughout much of North America, including south-central and midwestern United States. As most migratory monarchs traverse these areas during migration [16], migratory monarch populations may be particularly susceptible to changes in host plant identity in the summer breeding grounds [24]. Whether such changes result in positive or negative effects on monarch populations depends on the identity of the replacement species and the species being replaced and is difficult to predict from laboratory performance assays [44–45].

Monarch migrations may be determine by environmental characteristics, as they migrate north from March through May to avoid excessive heat and to track the emergence of young milkweed plants [17]. Indeed, the monthly distribution models of *Asclepias* and monarchs presented here are nearly identical to the monthly distribution models of monarchs presented by Batalden et al. [22]. However, there is growing evidence that much of the monarch migratory route may be genetically controlled. For example, monarchs navigate primarily using the sun as a directional guide, compensating for natural changes in the sun’s position over the course of the season [14,21]. As the migration is completed over multiple generations, flight paths are likely heritable among generations [21], although small-scale movements are almost certainly influenced by milkweed presence [14]. If large-scale patterns of monarch migrations are influenced less by *Asclepias* than by environmental cues, such as photoperiod, then monarchs may not exhibit the same northward expansion as *Asclepias*. If monarchs do match *Asclepias* range shifts, then they face longer southern migration distances in the fall (Fig. 9, [22]). Longer migration distances presumably negatively impact monarch fitness; fewer Atlantic coast migrations reach the overwintering ground in Mexico, which may be due to higher energy expenditure over a longer migration route [46].

It is worth noting that species distributions models are correlative, assume that the underlying data are unbiased, and that biotic interactions do not determine the extent of a species’ geographic range [40]. Although correlative, such models are useful in highlighting potential impacts of climate change on species’ distributions so that managers can begin making precautionary decisions to avoid population decline in the future [10]. MaxEnt is among the best-performing methods currently available for modeling species’ distributions [31], although models parameterized based on species physiology often perform better than correlative approaches like MaxEnt [47–48]. Data in this study were not collected following a systematic sampling routine and are geographically biased. However, species’ distribution models require only that environmental space be representatively sampled to provide accurate predictions of their ecological niches. I relied on citizen science data from Journey North supplemented with observations downloaded from GBIF. Spatial filtering to remove geographic bias from these data severely reduced sample size, limiting the predictive power of MaxEnt models. Furthermore, species-specific observations may have been biased or sparse. For example, many observations of *A*. *syriaca* were located in the midwestern United States, though it is quite common throughout most of the Atlantic coast and eastern United States as well. *Asclepias incarnata* was also primarily observed in the midwestern United States, although it is known to occur throughout the entire eastern United States. Further, small sample sizes of some species (*e*.*g*. *A*. *purpurascens*) limit the power of MaxEnt models. As such, many of these species-specific models should be interpreted with extreme caution.

Additionally, using models such as MaxEnt to predict future species distributions relies on climate change scenarios that are uncertain. To address this concern, I examined climate change as predicted under multiple GCMs and under two different emissions scenarios. In some cases, distribution models projected onto all four GCMs predict similar declines in *Asclepias* habitat suitability (*e*.*g*. in the Midwest), suggesting that this outcome is fairly likely. Finally, predicted habitat suitability based on climatic variables does not necessarily translate into a metric of *Asclepias* abundance. Urban areas and intensively managed farmland are unlikely to contain *Asclepias* at high densities [23]. Additionally, *A*. *incarnata* is typically restricted to wet soils near rivers and marshes, a habitat requirement not captured by these models. Given all of these assumptions and restrictions, my results do not provide a definitive indication of where *Asclepias* will occur in the future, but rather describe potential habitat suitability in uncertain future climates.

In summary, climate change may shift the optimal habitat of monarchs’ obligate *Asclepias* host plants further north, although these effects vary considerably among *Asclepias* species. The realized effects of climate change on monarch migrations depends on whether monarchs follow milkweed northward, whether current migratory routes experience a shift in *Asclepias* species identity, and whether monarch phenology matches that of *Asclepias* in future climates [22]. Compounded with habitat loss [23–25], my results suggest that monarch migrations and summer breeding grounds may undergo substantial changes in the future.

## Supporting Information

S1 Appendix Model results from different spatial filtering schemes. (PDF)

S1 Fig Spatially filtered observations used in MaxEnt models for each of the eight *Asclepias* species. (PDF)

S2 Fig Percentage variance explained by each environmental variable for each of the eight *Asclepias* species. (PDF)
