# Supplementary material for: Climate Change May Alter Breeding Ground Distributions of Eastern Migratory Monarchs (Danaus plexippus) via Range Expansion of Asclepias Host Plants
Source: PLoS One. 2015 Feb 23;10(2):e0118614. doi: 10.1371/journal.pone.0118614 (PMC4338007; doi:10.1371/journal.pone.0118614)
Supplement: S1 Appendix — (PDF) [file pone.0118614.s001.pdf]

Appendix 1 – Alternative Spatial Filtering Results

This appendix describes results from two alternative spatial filtering schemes: 600% spatial filtering and no filtering. Full results are presented, except for species-specific and monthly models, as those models were not possible under the largest spatial filtering scheme due to small sample sizes.

Section 1 – 600% Spatial Filtering (60 arc-minute resolution)

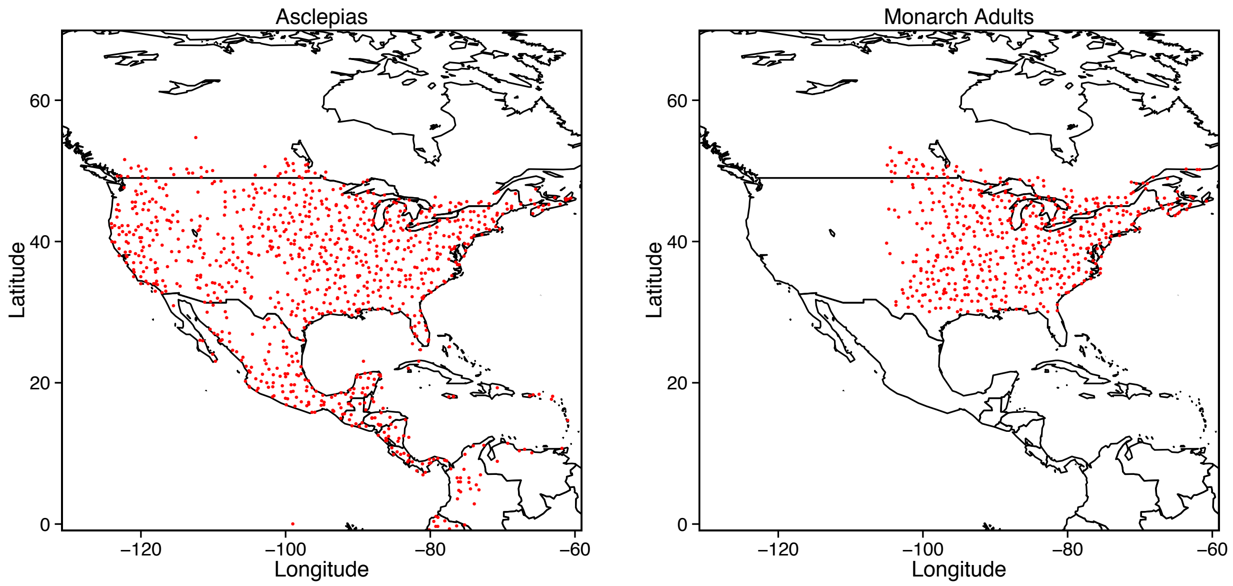

This spatial filtering scheme overlaid a 60 arc-minute grid over the data, choosing one random point from grids in a checkerboard pattern. This reduced the number of *Asclepias* records to 966 and the number of monarch records to 521.

Subsection 1.1 – *Asclepias* distribution

The 'All Variables' model best fit the data, similar to results presented in the main text. However, the model fit worse (AUC = 0.763) compared to the model in the main text (AUC = 0.805).

Model	AICc	n	AUC	ΔAICc
**All**	**18990.48**	**112**	**0.763**	**0**
Geographic	20127.63	62	0.564	1137.15
Cold	20777.90	57	0.753	1787.42
Warm	20811.96	36	0.755	1821.48
Precipitation	21523.58	54	0.703	2533.10

As in the main text, mean annual temperature accounted for more variance than any other predictor. Maximum temperature of the warmest month, mean temperature of the warmest quarter, and minimum temperature of the coldest month were also important predictors.

The predicted distribution of *Asclepias* was spottier than the results from the main text. The largest difference is the high probability of *Asclepias* in Louisiana and the low probability of *Asclepias* in Texas and Oklahoma, as well as the southeastern US (Fig. A1).

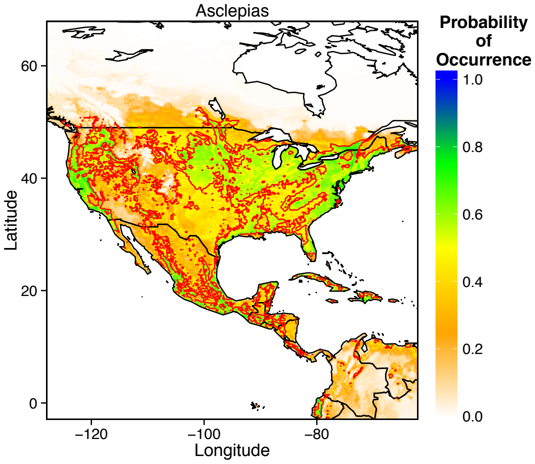

Figure A1. MaxEnt predictions of current *Asclepias* distribution given 600% spatial filtering (60 arc-minute resolution).

The largest difference is in the future predictions. Results from the main text indicate that *Asclepias* will occupy much of southern and eastern Canada under both climate change scenarios. Results from this filtering scheme (600%) are much different. Under moderate climate change, *Asclepias* occupies a very small portion of south-central Canada and the northeast US (as well as the west coast) (Fig. A2). Predictions of *Asclepias* distributions under severe climate change are similar to those from the main text, albeit with reduced occupancy predicted in the midwestern US and parts of western Canada (Fig. A2).

Subsection 1.2 – Monarch distribution

Model selection on the monarch data also indicated that the ‘Environment + Habitat’ model predicted monarch distributions best. This model also yielded similar goodness-of-fit to the model presented in the main text.

Model	AICc	n	AUC	ΔAICc
**Environment and Habitat**	**9805.81**	**59**	**0.887**	**0**
All	9878.84	94	0.886	73.02
Habitat	10333.65	16	0.795	527.83
Environment	10651.54	62	0.889	845.73
Geographic	11083.48	46	0.680	1277.67

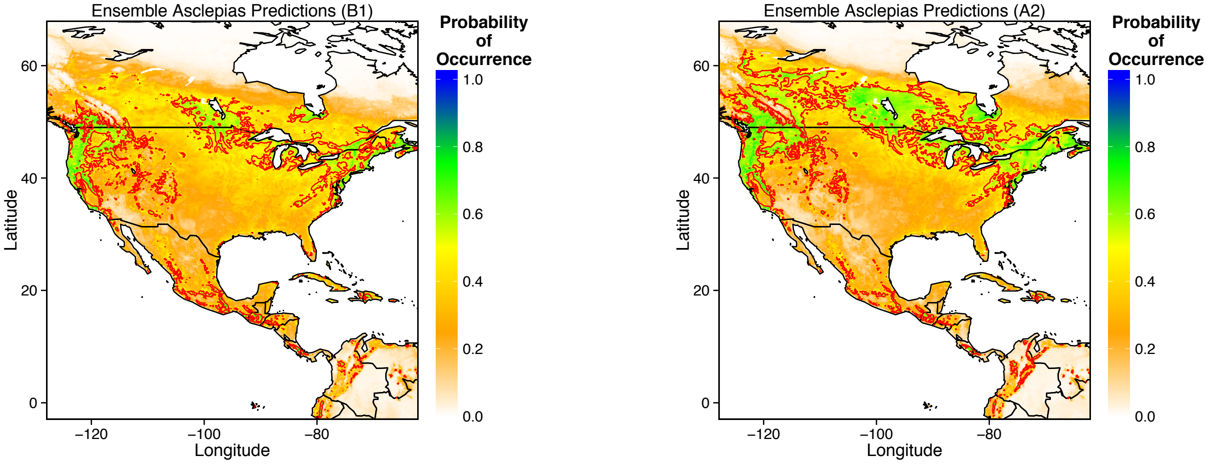

Figure A2. Predicted distribution of *Asclepias* under two climate change scenarios resulting from a spatial filtering of 600% (60 arc-minute resolution).

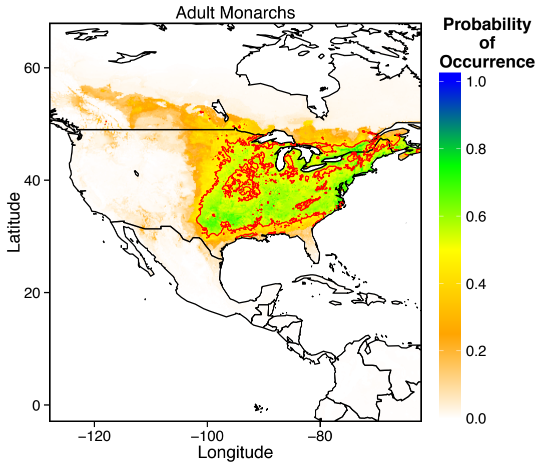

Figure A3. MaxEnt predictions of current monarch distribution given 600% spatial filtering (60 arc-minute resolution).

However, in this model, temperature seasonality was as important a predictor of monarch observations as *Asclepias* distribution, each accounting for ~30% of the variance in monarch sightings. Precipitation of the warmest quarter, mean annual temperature, annual precipitation, and mean temperature of the warmest quarter were also important predictors of monarch observations.

As in the main text, eastern migratory monarchs were predicted to occur throughout most of the central and eastern US, with a northern limit around the Great Lakes region. These results are largely consistent with those in the main text, except reduced probability of occurrence for monarchs along the Gulf Coast and patches of low-probability areas in the midwestern US.

However, as with *Asclepias*, projections of monarch distributions into the future differed dramatically for this spatial filtering scheme. The model presented in the main text predicted that monarchs will occur throughout the central, northeastern, and midwestern US and throughout southern and eastern Canada. In contrast, projections of monarch distributions into the future from the filtering scheme here did not extent monarch distributions northward into Canada at all (Fig. A4).

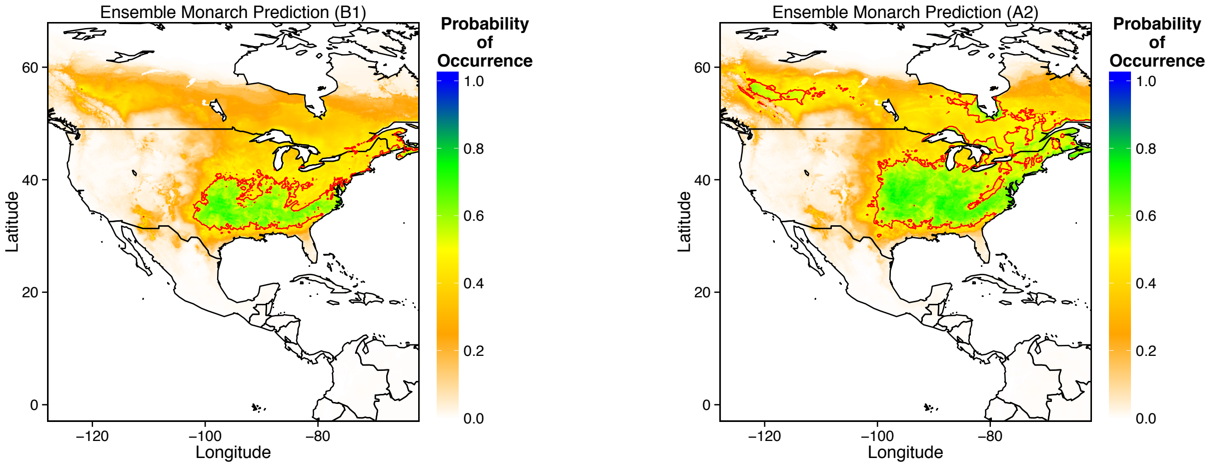

Figure A4. Predicted distribution of monarch adults under two climate change scenarios resulting from a spatial filtering of 600% (60 arc-minute resolution).

Section 2 – No Spatial Filtering

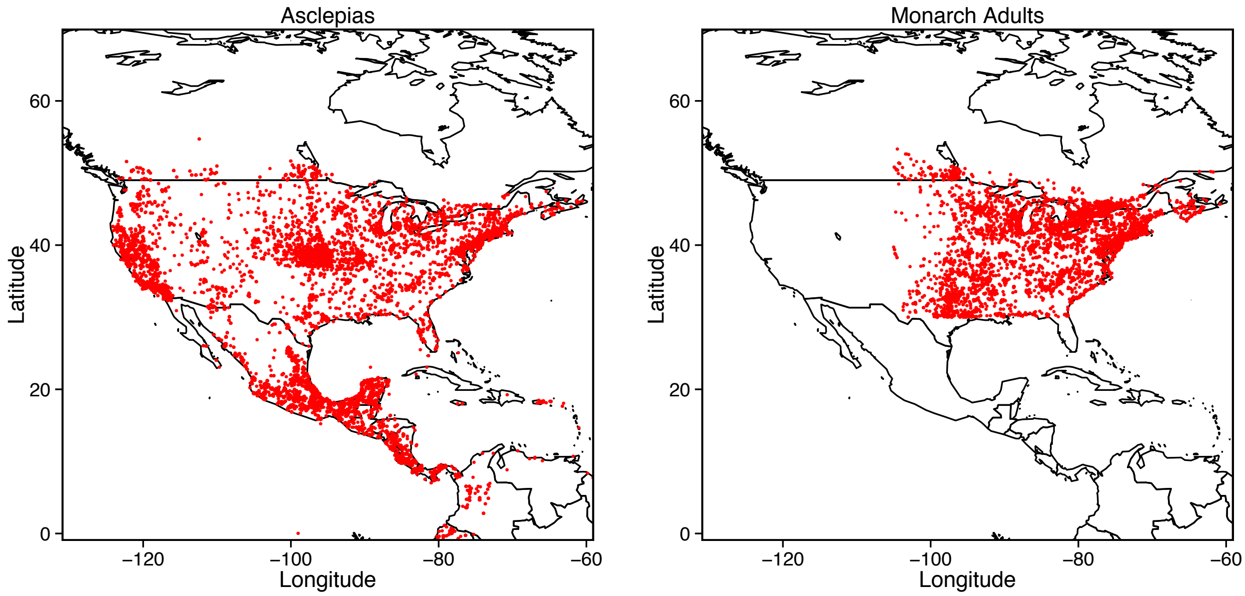

Models presented here did not use any spatial filtering, instead using all 8,606 monarch observations and 12,983 *Asclepias* observations.

Subsection 2.1 – *Asclepias* distribution

As in other filtering schemes, the 'All Variables' model provided the best fit to the overall *Asclepias* distribution. As expected given the increased sample size, the goodness-of-fit for this model (AUC = 0.895) was substantially higher than the model presented in text (AUC = 0.805).

Model	AICc	n	AUC	ΔAICc
**All**	**244910.37**	**201**	**0.895**	**0**
Geographic	267340.75	109	0.706	22430.38
Cold	271031.11	128	0.863	26120.74
Warm	276597.70	78	0.802	31687.32
Precipitation	279981.97	106	0.822	35071.60

Mean annual temperature again explained > 50% of the variance, while minimum temperature of the coldest month and annual precipitation also accounted for ~ 15% of the variance each.

However, the model demonstrated considerable overfitting, rarely predicting areas outside of the observed locations to be suitable for *Asclepias*. For example, most of the US was predicted as unsuitable for *Asclepias*, except small portions of the west coast, Midwest, and northeast (Fig. A5). This does not represent the known distribution of *Asclepias*, which ranges throughout the midwestern, central, and southeastern US. Instead, this more likely represents the distribution of sampling intensity.

Projections of *Asclepias* distributions under two climate change scenarios yielded somewhat similar results to those of the main text. *Asclepias* extended its northern range, but to a lesser degree than the results from the main text, rarely extending beyond the Great Lakes region (Fig. A6). Under severe climate change, only a narrow area around the Great Lakes and northeastern US was projected to be suitable for *Asclepias.*

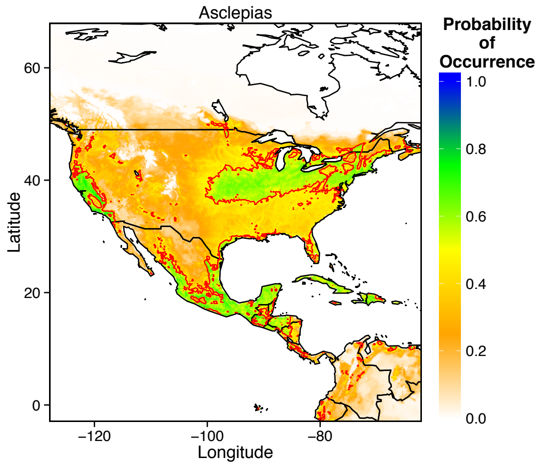

Figure A5. MaxEnt predictions of current *Asclepias* distribution without any spatial filtering.

Subsection 1.2 – Monarch distribution

As in the other filtering schemes, the 'Environment + Habitat' model provided the best fit to monarch distributions.

Model	AICc	n	AUC	ΔAICc
**Environment and Habitat**	**153976.16**	**116**	**0.948**	**0**
All	154077.48	130	0.949	101.32
Habitat	165679.30	41	0.854	11703.14
Environment	167720.59	123	0.939	13744.43
Geographic	182958.11	80	0.753	28981.96

The predicted *Asclepias* distribution accounted for ~ 50% of the variance in monarch observations. Temperature seasonality and precipitation of the warmest quarter also explained a substantial fraction of the variance in monarch observations.

However, as with *Asclepias*, the predicted monarch distribution did not extend beyond sampled locations and thus represented sampling intensity more than habitat suitability. For instance, monarchs were predicted to occur only in the Great Lakes region and a small portion of eastern Texas (Fig. A7).

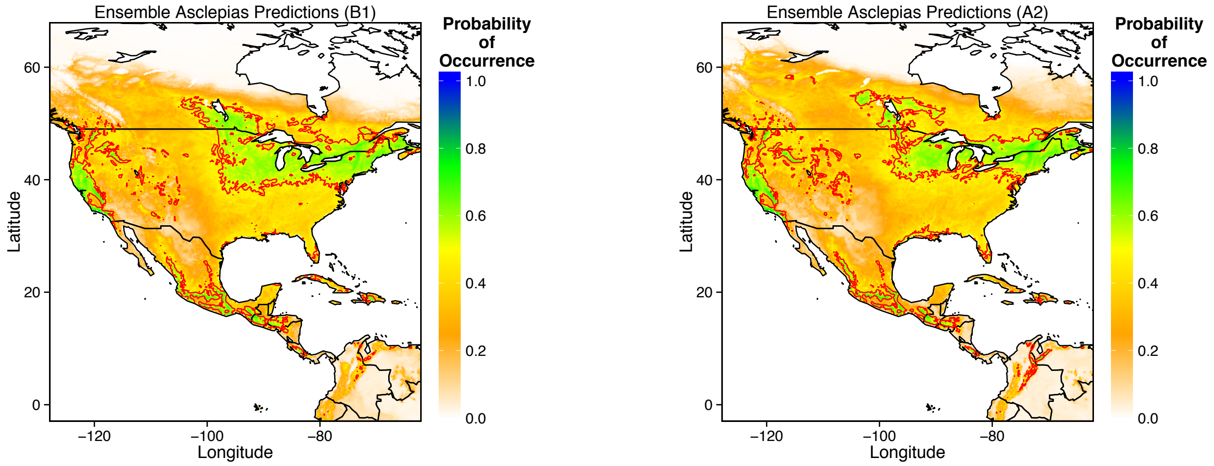

Figure A6. Predicted distribution of *Asclepias* under two climate change scenarios without spatial filtering.

In both climate change scenarios, models without spatial filtering predicted that monarchs would only occur in eastern and southern Canada (Fig. A8). The southern limit of monarchs extended no further south than the Great Lakes in either scenario.

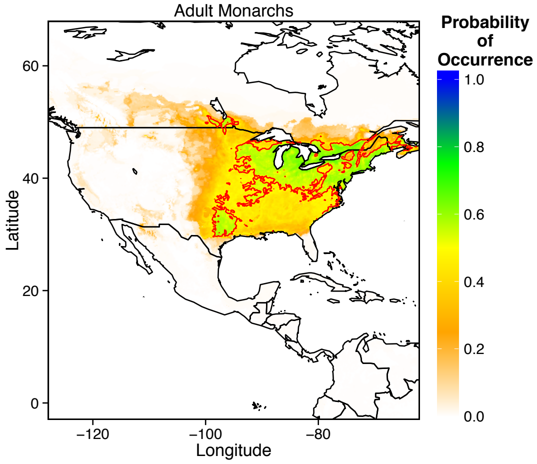

Figure A7. MaxEnt predictions of current monarch distribution without any spatial filtering.

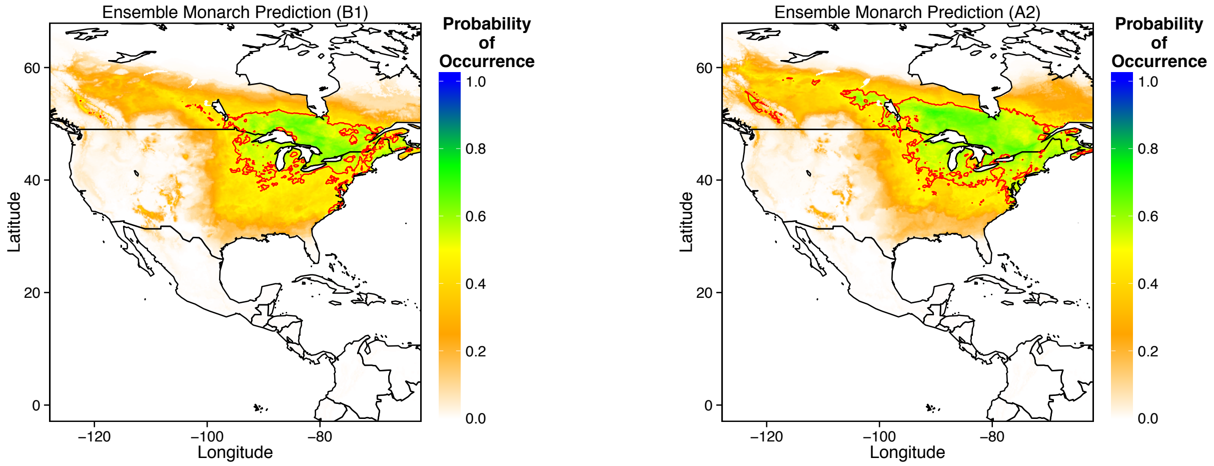

Figure A8. Predicted distribution of adult monarchs under two climate change scenarios without spatial filtering.
