# Supplementary figures and images for: Climate Change May Alter Breeding Ground Distributions of Eastern Migratory Monarchs (Danaus plexippus) via Range Expansion of Asclepias Host Plants
Source: PLoS One. 2015 Feb 23;10(2):e0118614. doi: 10.1371/journal.pone.0118614 (PMC4338007; doi:10.1371/journal.pone.0118614)

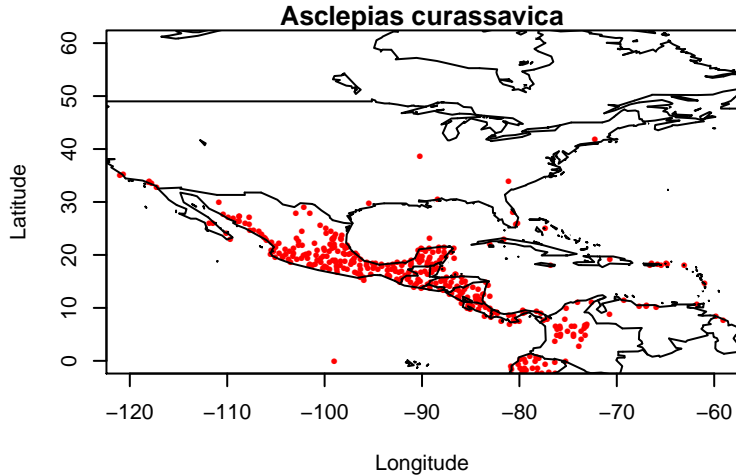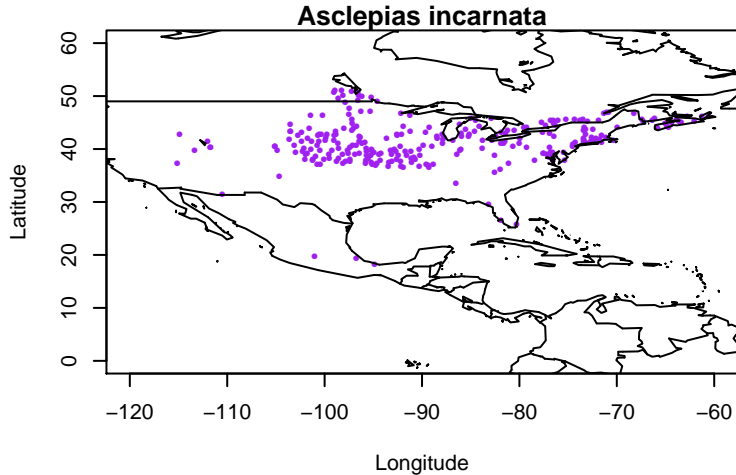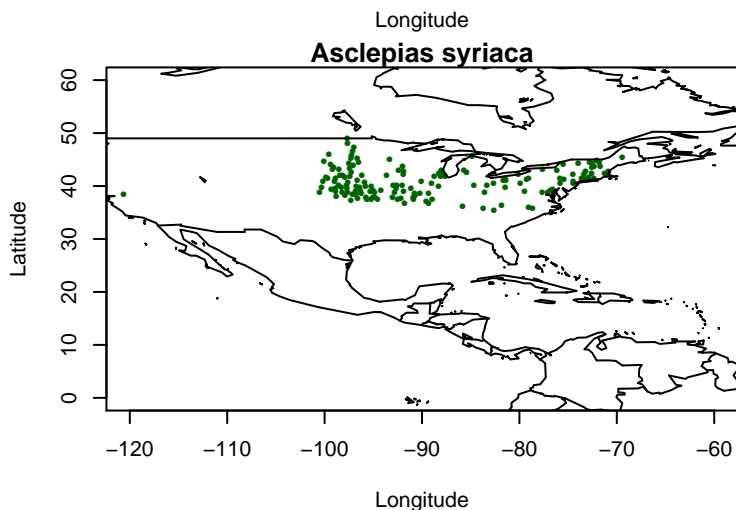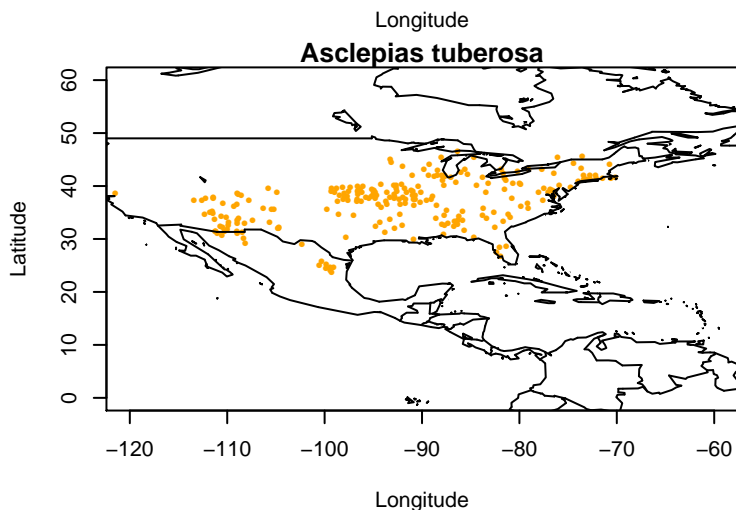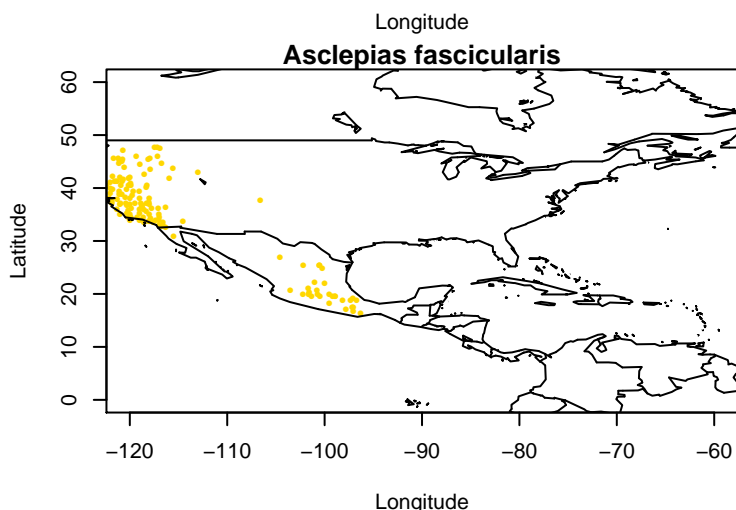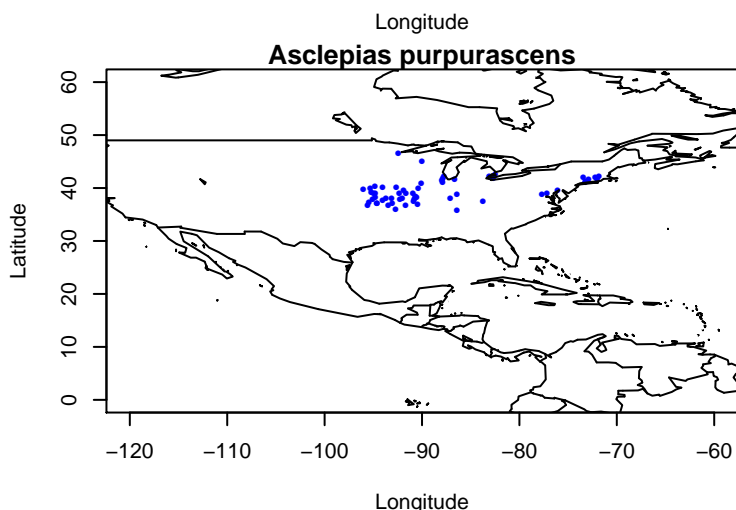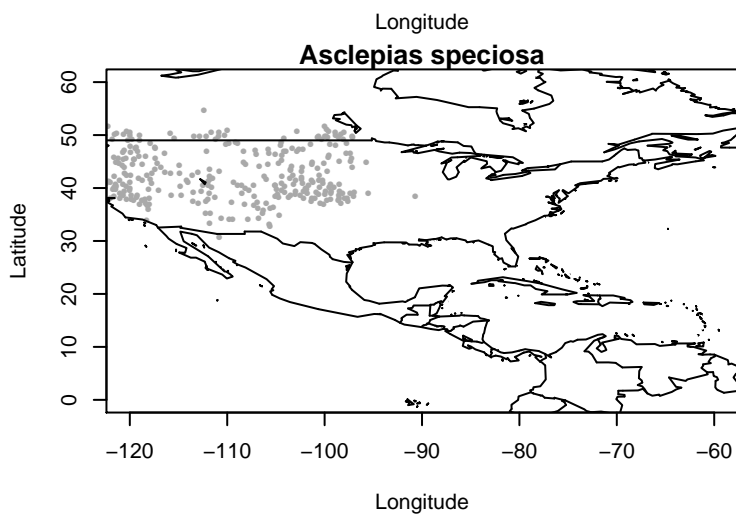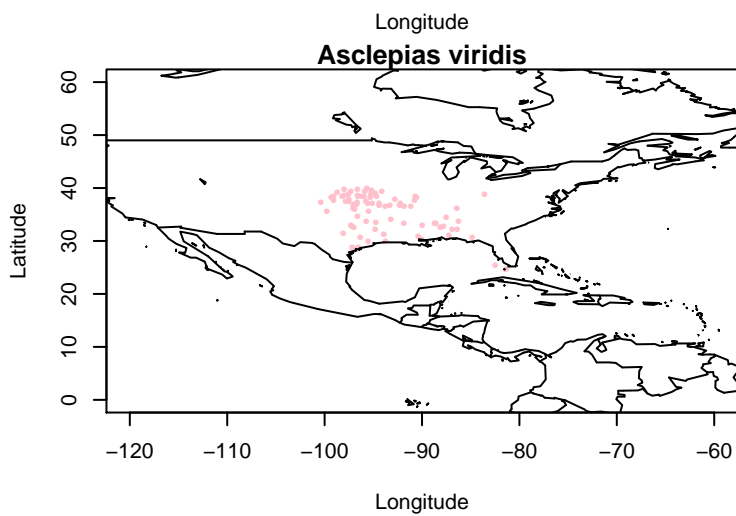

Supplement: S1 Fig — (PDF) [file pone.0118614.s002.pdf]
