# Supplementary material for: Climate Change May Alter Breeding Ground Distributions of Eastern Migratory Monarchs (Danaus plexippus) via Range Expansion of Asclepias Host Plants
Source: PLoS One. 2015 Feb 23;10(2):e0118614. doi: 10.1371/journal.pone.0118614 (PMC4338007; doi:10.1371/journal.pone.0118614)
Supplement: S2 Fig — (PDF) [file pone.0118614.s003.pdf]

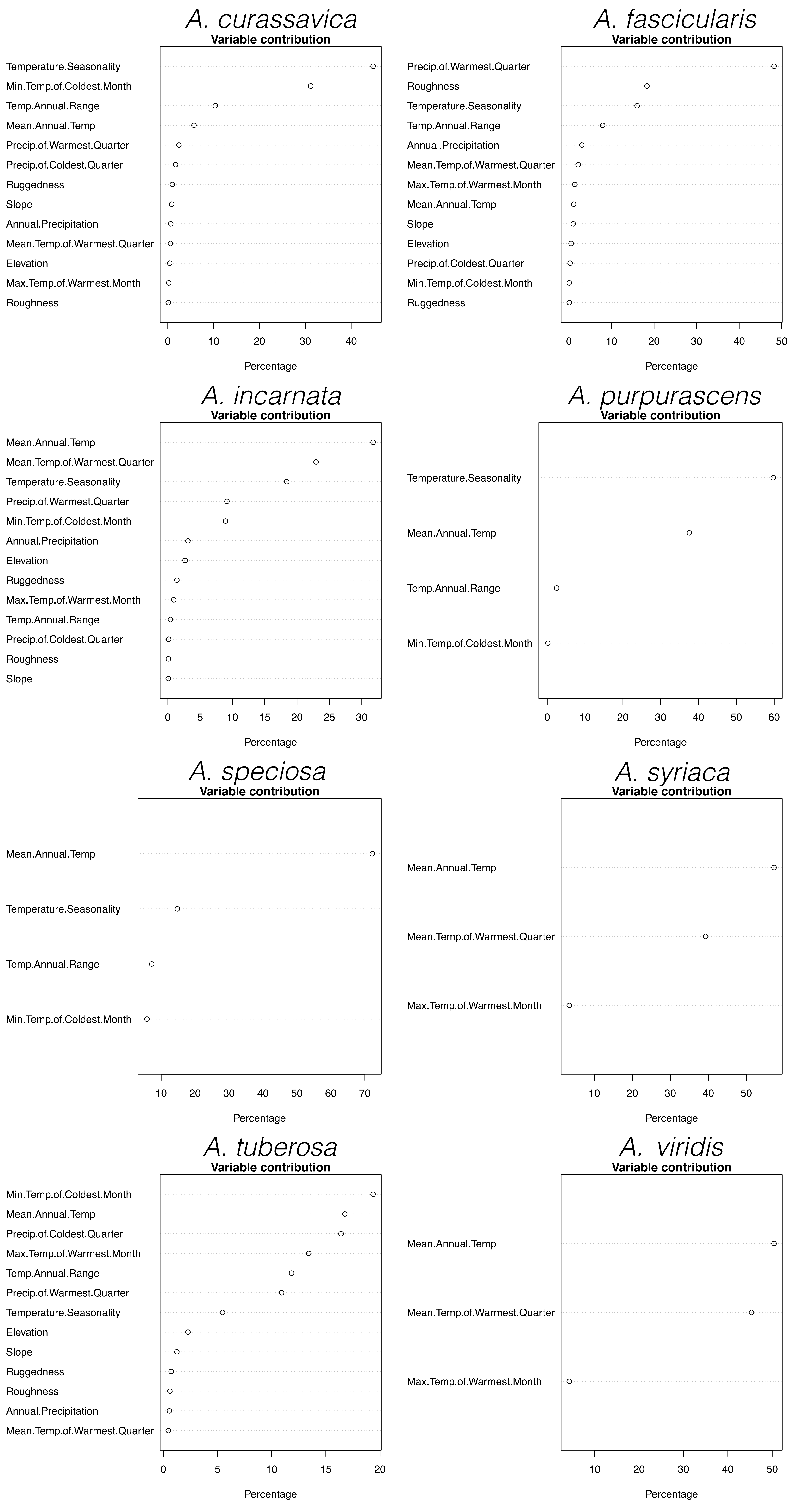
A. curassavica
Variable contribution
Temperature.Seasonality
Min.Temp.of.Coldest.Month
Temp.Annual.Range
Mean.Annual.Temp
Precip.of.Warmest.Quarter
Precip.of.Coldest.Quarter
Ruggedness
Slope
Annual.Precipitation
Mean.Temp.of.Warmest.Quarter
Elevation
Max.Temp.of.Warmest.Month
Roughness
0
10
20
30
40
Percentage
A. fascicularis
Variable contribution
Precip.of.Warmest.Quarter
Roughness
Temperature.Seasonality
Temp.Annual.Range
Annual.Precipitation
Mean.Temp.of.Warmest.Quarter
Max.Temp.of.Warmest.Month
Mean.Annual.Temp
Slope
Elevation
Precip.of.Coldest.Quarter
Min.Temp.of.Coldest.Month
Ruggedness
0
10
20
30
40
50
Percentage
A. incarnata
Variable contribution
Mean.Annual.Temp
Mean.Temp.of.Warmest.Quarter
Temperature.Seasonality
Precip.of.Warmest.Quarter
Min.Temp.of.Coldest.Month
Annual.Precipitation
Elevation
Ruggedness
Max.Temp.of.Warmest.Month
Temp.Annual.Range
Precip.of.Coldest.Quarter
Roughness
Slope
0
5
10
15
20
25
30
Percentage
A. purpurascens
Variable contribution
Temperature.Seasonality
Mean.Annual.Temp
Temp.Annual.Range
Min.Temp.of.Coldest.Month
0
10
20
30
40
50
60
Percentage
A. speciosa
Variable contribution
Mean.Annual.Temp
Temperature.Seasonality
Temp.Annual.Range
Min.Temp.of.Coldest.Month
10
20
30
40
50
60
70
Percentage
A. syriaca
Variable contribution
Mean.Annual.Temp
Mean.Temp.of.Warmest.Quarter
Max.Temp.of.Warmest.Month
10
20
30
40
50
Percentage
A. tuberosa
Variable contribution
Min.Temp.of.Coldest.Month
Mean.Annual.Temp
Precip.of.Coldest.Quarter
Max.Temp.of.Warmest.Month
Temp.Annual.Range
Precip.of.Warmest.Quarter
Temperature.Seasonality
Elevation
Slope
Ruggedness
Roughness
Annual.Precipitation
Mean.Temp.of.Warmest.Quarter
0
5
10
15
20
Percentage
A. viridis
Variable contribution
Mean.Annual.Temp
Mean.Temp.of.Warmest.Quarter
Max.Temp.of.Warmest.Month
10
20
30
40
50
Percentage
